# Antioxidant Clove Extract Inhibits Lipid Droplet Accumulation and Lipid Oxidation in Hepatocytes

**DOI:** 10.3390/metabo16010007

**Published:** 2025-12-22

**Authors:** Satomi Monde, Dya Fita Dibwe, Shion Iwasaki, Shu-Ping Hui

**Affiliations:** 1 Graduate School of Health Sciences, Hokkaido University, Kita-12, Nishi-5, Kita-Ku, Sapporo 060-0812, Japan; monde.satomi.p3@elms.hokudai.ac.jp (S.M.); iwasaki.shion.e7@elms.hokudai.ac.jp (S.I.); 2 Faculty of Health Sciences, Hokkaido University, Kita-12, Nishi-5, Kita-Ku, Sapporo 060-0812, Japan; dibwedf@hs.hokudai.ac.jp

**Keywords:** plant metabolomics, secondary metabolites, NMR, bioactive compounds, functional foods, nutraceuticals, lipid droplet accumulation inhibition, antioxidant activity index

## Abstract

Background: Recent studies have shown that plant-based dietary extracts can prevent the formation of lipid droplets (LDs) and oxidized lipid droplets (oxLDs) in liver cells. These results indicate that these extracts might be useful in addressing metabolic dysfunction-associated fatty liver disease (MAFLD) and its more severe form, metabolic dysfunction-associated steatohepatitis (MASH). In our ongoing study, we evaluated the potential of various food extracts to inhibit the accumulation and oxidation of LDs in liver cells to prevent metabolic MAFLD and MASH. Methods: The antioxidant activity index was determined using the DPPH assay, cell viability was assessed via cytotoxicity and lipotoxicity, and lipid droplet accumulation inhibition (LDAI) assays were performed. Metabolome analysis was performed using 1D-NMR [^1^H, ^13^C, DEPT 90, and 135] techniques. Results: Dietary clove (*Syzygium aromaticum*) extract exhibited antioxidant properties and inhibited linoleic acid-induced lipid droplet (LD) accumulation (LDA) and oxidized LDA (oxLDA) in HepG2 cells. Additionally, an analysis of the metabolome of dietary clove bioactive LDAI using 1D-NMR showed that clove extract (CE) mainly consists of hydroxybenzoic acids (HBAs) and hydroxycinnamic acids (HCAs), along with minor amounts of carbohydrates, coumarins, polyphenolic compounds, and small quantities of polyols, fatty acyls, small peptides, and amino acids. This suggests that CE could be a promising resource for developing functional foods and nutraceuticals and discovering drugs for treating MAFLD, MASH, and related conditions.

## 1. Introduction

Metabolic dysfunction-associated fatty liver disease (MAFLD), the most prevalent chronic liver condition globally, is characterized by its potential to progress to liver fibrosis. The underlying pathological mechanisms of MAFLD are complex and have not yet been fully elucidated. Insulin resistance and lipid accumulation, which are collectively associated with lipotoxicity, are central to its pathogenesis. These elements cause different causative agents to release free radicals, which in turn cause lipid peroxidation, necroinflammation, fibrosis, and disease progression. Uncertainty surrounds the exact pathomechanism of MAFLD [1,2,3]. The management of co-existing metabolic conditions, such as hyperglycemia and hyperlipidemia, and lifestyle changes, such as dietary changes and increased physical activity, are the main approaches for treating MAFLD, which is particularly difficult to treat. The FDA’s recent approval of resmetirom is a major step forward, although lifestyle modifications are the only proven treatments for MAFLD. Additionally, antioxidants derived from natural sources offer potential alternative treatment avenues, and secondary metabolites, also known as natural products, show considerable promise as therapeutic agents for MAFLD [4,5,6,7,8,9,10,11].

Natural products in functional foods and nutraceuticals can reduce the accumulation of lipid droplets (LDs) and oxidized lipid droplets (oxLDs) by influencing the levels of neutral lipid triglycerides (TAG) and lipid hydroperoxide [TAG-(OOH)_n_] in liver cells, which can reduce MAFLD and metabolic dysfunction-associated steatohepatitis (MASH) [12,13,14,15]. Previous research has demonstrated the significant potential of these easily accessible natural products in drug development and the prevention of MAFLD/MASH. A promising approach for preventing MAFLD and developing new therapeutic medications is the recent scientific investigation of how natural plant products affect the production of lipid hydroperoxides and lipid droplets in liver cells, in conjunction with metabolome analysis [10,11,12,13,14]. Biologically active compounds that inhibit lipid droplet accumulation (LDA) in liver cells are emerging as potential treatment options for MAFLD/MASH. In this context, our previous study identified flazine, an alkaloid belonging to the β-carboline class, characterized by a carboxyl group at the C-3 position and a furfuryl alcohol group at the C-1 position, along with its piperidine C-ring derivative isolated from oysters. These findings highlight the critical role of plant-based food extracts and β-carboline alkaloids in MAFLD prevention through LDA inhibition. This suggests that nutraceuticals and functional foods may harbor valuable LDAI candidates that could prove beneficial in managing chronic liver conditions, such as MAFLD/MASH. Furthermore, we recently examined common vegetable extracts from Hokkaido-grown garlic and beans and recognized their potential as functional food sources because of their capacity to prevent lipid accumulation and reduce the formation of lipid droplets (LDs) in cellular studies. Studies on hepatocytes have shown that these extracts influence lipid droplet accumulation (LDA) and lipid oxidation. Additionally, natural bioactive extracts sourced from clove have been shown to prevent lipid accumulation in HepG2 cells. Studies on hepatocytes can provide scientific evidence that these extracts influence lipid droplet accumulation (LDA), lipid oxidation, and health promotion.

**Figure 1:**
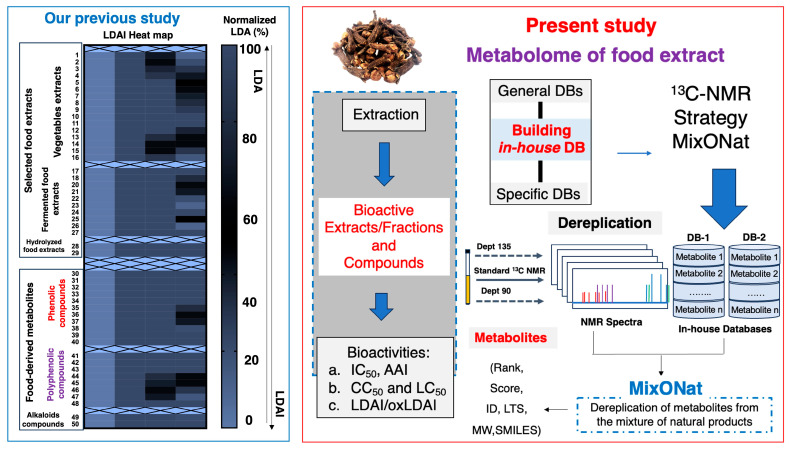
Previous studies of LDAI activity involved the screening of food extracts (black: vegetables, fermented and hydrolyzed) and compounds (red: phenolic compounds and purple: polyphenolic compounds), and the present study involved the metabolomic analysis of LDAI/oxLDAI clove food extracts.

Recent research indicates that the extracts from plant-based foods and their respective secondary metabolites possess LDAI properties, showing their efficiency in the management of LD/oxLD accumulation and also expressing significant LDAI/oxLDAI activities. Plant extracts and their bioactive compounds with LDAI properties decrease intracellular TAG species, enhance the rate of fat catabolism, and inhibit de novo lipid synthesis. The emerging principle method for the treatment of hepatic steatosis is the reduction in both LDA levels and lipid peroxidation. The study aimed at the identification of potential compounds and food extracts capable of preventing MAFLD and MASH. This is performed by evaluating the efficiency of various dietary extracts based on their antioxidant properties and ability to prevent LD accumulation (LDAI) in HepG2 cells exposed to linoleic acid (LA). During the continuing study into the screening of LDAI/oxLDAI activities of food extracts, dietary extracts from clove (*Syzygium aromaticum*) were identified as notable bioactive LDAI candidates. The clove extract showed significant antioxidant properties and importantly inhibited both the accumulation of LDs and the formation of oxLDA in HepG2 cells when induced by linoleic acid, a common fatty acid implicated in lipotoxicity and lipid oxidation. In this study, plant metabolomic fingerprinting and rapid dereplication of the chemical constituents present in an identified bioactive clove extract and its fractions were performed using advanced 1D-NMR techniques, specifically ^1^H, ^13^C, and DEPT (90 and 135) analyses. The systematic metabolomic investigation of the fractions of the bioactive clove extract may show the presence of chemo-specific constituents in their metabolomes, explaining the expressed LDAI/oxLDAI activities (Figure 1). The outcome of the current study may serve of important foundation for further in-depth drug discovery and nutraceutical studies for the prevention of MAFLD.

## 2. Materials and Methods

### 2.1. Chemical and Extraction

Oleic and linoleic acids were utilized for cell-free fatty acid loading. The mobile phase for liquid chromatography/mass spectrometry (LC/MS) consisted of ammonium acetate from Wako Pure Chemical (Osaka, Japan) and LC-grade methanol from Kanto Chemical (Tokyo, Japan). A clove sample was collected in April 2024 from the Shinjuku marketplace by D. F. D. The bud part of the clove was used for extraction in this study. Based on the strict regulation of food used in Japan, the confirmation of botanical authentication was verified, and the voucher specimen number HU-FHS-HIC-LALA_00221 was stored at the Health Innovation Center of the University’s Health Science Faculty. Comprehensive details regarding the chemicals and equipment employed in this study are available in the Supplementary Materials, specifically in Supplementary File S1, as previously documented [10,13,14].

### 2.2. Extraction and Fractionation of Clove Extract

The CE powder (28.01 g) was extracted with MeOH under sonication (2 L, 90 min, 3**×**) at room temperature, and the solvent was evaporated under reduced pressure to obtain the MeOH extract (8.53 g). The MeOH extract was chromatographed on column silica gel using an n-hexane–EtOAc and CHCl_3_-MeOH solvent system with increasing polarity to yield 15 fractions (F1–F15) [F1: n-hexane–EtOAc (85:15) eluate; 17.1 mg; F2: n-hexane–EtOAc (75:25) eluate (1462.8 mg); F3, n-hexane–EtOAc (65:35) eluate, 1327.3 mg; F4: n-hexane–EtOAc (50:50) eluate (266.5 mg); F5: n-hexane–EtOAc (40:60) eluate (84.7 mg); F6: n-hexane–EtOAc (40:60) eluate (112.8 mg); F7: n-hexane–EtOAc (30:70) eluate, 65.8 mg fraction; F8: n-hexane–EtOAc (30:70) eluate (33.7 mg); F9: n-hexane–EtOAc (20:80) eluate, 62.5 mg; F10: n-hexane–EtOAc (20:80) eluate (54.8 mg); F11: n-hexane–EtOAc (10:90) eluate, 55.4 mg; F12: n-hexane–EtOAc (0:100) eluate (125.7 mg); F13: CHCl_3_-MeOH (10:70) eluate, 34.8 mg; F14: CHCl_3_-MeOH (20:70) eluate, 1802.0 mg; F15: CHCl_3_-MeOH (30:70) eluate, 1235.6 mg].

### 2.3. Antioxidant Activity Index, Evaluation of Cell Viability, and Lipid Droplet Accumulation Inhibition

The antioxidant activity index (AAI) was evaluated using the DPPH radical assay, following previously established methodologies [13,14]. A 10 mg DPPH sample was dissolved in 100 mL of ethanol to create a working solution. Various extract concentrations ranging from 6.25 to 200 μg/mL were generated through serial two-fold dilutions. In a 96-well plate, 100 μL of each diluted extract was combined with an equivalent volume of DPPH working solution for the assay. The absorbance was measured at 517 nm after incubating the samples for 30 min at room temperature, protected from light. The reference standards for comparison were chlorogenic and ascorbic acids (vitamin C). The following formula was used to calculate the percentage of DPPH radical scavenging activity (%RSA): %RSA = [(Acontrol − Asample)/Acontrol] × 100, where ‘A’ is the absorbance measured at 517 nm. Based on the AAI values, the antioxidant activity was categorized using the criteria of Scherer and Godoy as poor (AAI < 0.5), moderate (0.5 ≤ AAI ≤ 1.0), strong (1.0 < AAI ≤ 2.0), and very strong (AAI > 2.0).

### 2.4. Evaluation of Cell Viability

In-depth analyses of lipid accumulation and cellular reactions to toxic agents were performed using specific biological assays, such as cytotoxicity and lipotoxicity tests. HepG2 cells, a known cell line, were the specific target of the experimental techniques used in this study. The RIKEN BRC Cell Bank in Ibaraki, Japan, is the source of the HepG2 cells. The cytotoxicity and lipotoxicity tests were performed according to the detailed procedural instructions provided by the manufacturer of the reagents or cells. Furthermore, as the accompanying references [10,13,14] demonstrate, the methodology used in these investigations was in line with and expanded upon the previously published studies.

### 2.5. Lipid Droplet Accumulation Inhibition

An Oil Red O assay was used to measure the LDAI activity. Each treatment group had four replicates, and the assay was performed in 24-well plates. Briefly, Oil Red O staining was performed as described below. The culture media were removed, and the cells were washed twice with 200 μL of PBS. Subsequently, 200 μL of 10% formalin was added to each well and incubated for 10 min to fix the cells. After removing the formalin and washing twice with 200 μL of PBS, 200 μL of 60% isopropanol (IPA) was added and incubated for 10 min. The 60% IPA was then removed, and Oil Red O working solution (prepared by mixing Oil Red O stock solution with distilled water at a 6:1 ratio, followed by filtration) was added and incubated for 15 min. After staining, the dye was removed, the wells were washed once with 200 μL of 60% IPA, and then washed twice with 300 μL of phosphate-buffered saline (PBS). For quantification, 200 μL of 100% IPA was added to elute the bound dyes. From this extract, 150 μL of the supernatant was transferred to a 96-well plate, and the absorbance at 560 nm was measured using an ARVO-MX microplate reader. (LDAI): %LDAI = [(Asample − ABlank)/Acontrol LA(+)] × 100, where ‘A’ is the absorbance measured. LDA formation was evaluated from %control LA(+)] − %control LA(−)]. The LDAI value was normalized with Acontrol LA(+).

### 2.6. Fluorescence Staining Assay

Staining with Oil Red O was first partially performed according to the previously described method in Section 2.5 [10,13,14] and then modified in the procedure described below for fluorescence staining. Liperfluo was dissolved in serum-free medium at a concentration of 112.5 μM. A 1:1000 dilution of serum-free medium containing Liperfluo and Hoechst solution was prepared. The mixture was applied to cells that had been subjected to Oil Red O staining, and the cells were incubated in the dark for 30 min. Images were taken with a BZ-9000 fluorescence microscope (Keyence Co., Ltd., Osaka, Japan). TRITC and DAPI-B filters were purchased from Keyence Corporation (Osaka, Japan).

### 2.7. Metabolite Profiles of Clove Based on ^1^H-NMR Analysis

Nuclear magnetic resonance (NMR) analysis was used to examine the metabolite profiles of clove. Refer to the Supplementary Materials (Supplementary File S1) for comprehensive details on the technical aspects and methods of metabolite profiling using NMR.

### 2.8. Rapid Dereplication of Secondary Metabolites from Clove Metabolome Using 1D-NMR

Numerous 1D-NMR experiments, such as ^1^H-NMR, ^13^C-NMR, DEPT-135, and DEPT-90, were performed using a JEOL ECX400 NMR spectrometer (Tokyo, Japan), The chemical shifts, denoted as δH and δC, were quantified in parts per million (ppm). The experimental procedure involved dissolving a 30 mg sample of the methanol extract in 600 μL of CDCl_3_. The ^13^C-NMR spectra were acquired at a frequency of 100 MHz. Data processing was performed using the JEOL software version 6.3, specifically the Delta NMR Processing and software package. Spectral calibration was achieved by referencing the characteristic solvent peaks of CDCl_3_, which appeared at δC = 77.16 ppm. The analytical process encompassed manual phasing and baseline correction, followed by the alignment of the DEPT experiments with the corresponding ^13^C spectra using a predefined δC value for consistency. The chemical shifts, denoted as δH and δC, were quantified in parts per million (ppm). Simultaneously, an auxiliary software tool named MixONat (v. 1.0.1) was created by the SONAS lab at Université Angers in France [15]. This software is specifically engineered for the dereplication of natural product mixtures using ^13^C NMR spectroscopy. MixONat systematically aligns the δC values of natural products identified within mixtures with a curated database, meticulously considering their respective signal multiplicities. Experimental data obtained from the ^13^C-NMR, DEPT-90, and DEPT-135 spectra were exported in Microsoft Excel format (version 16.45) as reference.csv files for further analysis. These files were subsequently used as the input for the MixONat software. The reference.csv file structure is organized such that the δC values are listed in descending order, with their associated intensities recorded on the same line and separated by commas. This software is specifically engineered for the dereplication of natural product mixtures using ^13^C NMR spectroscopy. The software uses a large dataset of molecular structures from internal structural databases to process the data. These include Lamiaceae DB-1, comprising 980 molecules; DB2, the Myrtaceae DB, containing 2217 molecules; and DB3, the Euphorbiaceae DB, containing 6286 molecules. These databases contain ^13^C-NMR data pertaining to natural products sourced from the scientific literature via LOTUS DB. MixONat generates compound proposals by assigning each a score on a scale of 0–1 (equivalent to 0–100%), where a score of 1 signifies an exact match and a score of 0 indicates no similarity to any compounds within the database. Tentative compound identifications were made based on scores exceeding a threshold of 0.70. Following this initial identification, the experimental data for the natural products that received the highest scores were compared with the established literature data for validation, as detailed in the Supplementary Materials.

## 3. Results

### 3.1. Cell Viability and LDAI Activity of Clove Extract (CE)

**Figure 2:**
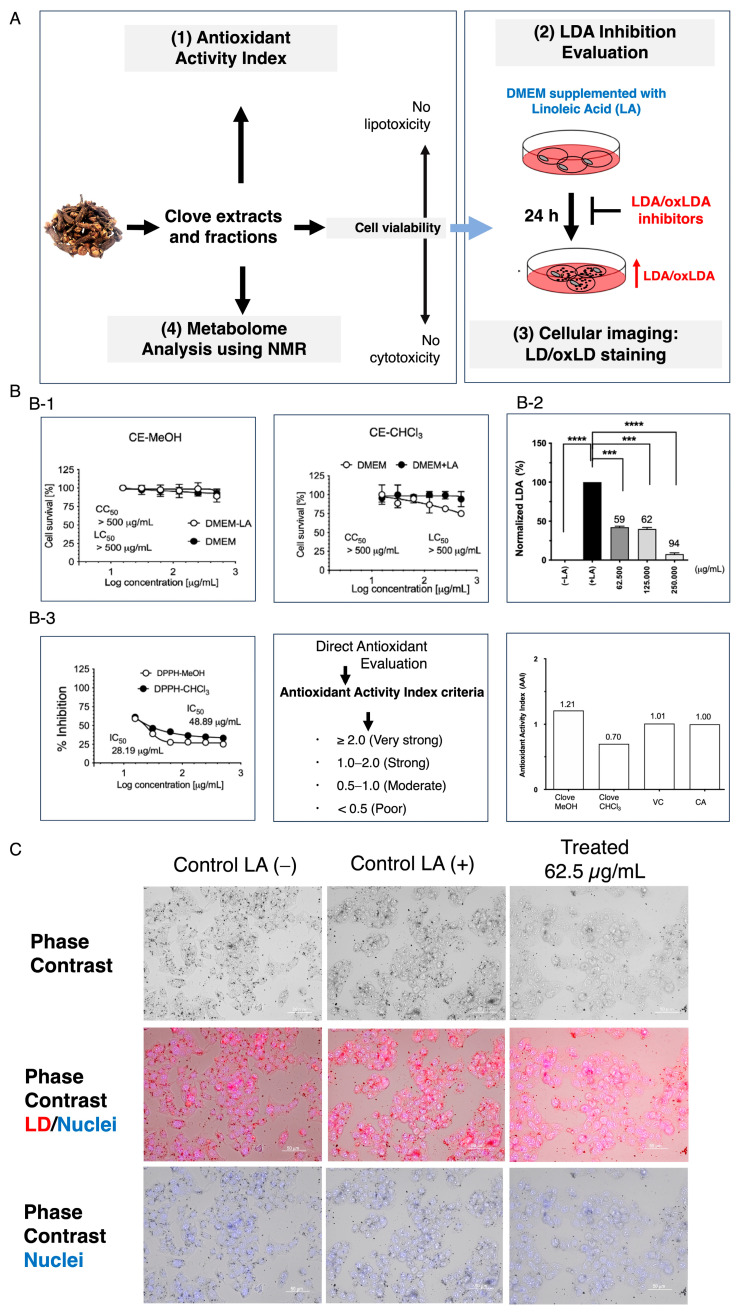
(**A**) Methodology for identifying food-derived antioxidants and substances that inhibit lipid droplet accumulation. (1) Evaluation of direct antioxidant properties and antioxidant activity index. (2) Assessment of LDAI/oxLDAI under fatty acid loading conditions in relation to Keap1-Nrf2 pathway regulation. (3) Cellular imaging: LD/oxLD staining. (4) Metabolome analysis was performed using NMR. (**B**) (**B-1**) Cytotoxicity and lipotoxicity of clove extracts (CE-MeOH and CE-CHCl_3_) in HepG2 cells. Cytotoxicity (CC_50_) was expressed as the concentration at which 50% of the cells died in DMEM without fatty acids (−LA). Lipotoxicity (LC_50_) was expressed as the concentration at which 50% of the cells died, especially in DMEM loaded with fatty acids (+LA). (**B-2**) The graph illustrates the mean LDAI/oxLDAI values (four replicates) for CE-MeOH in LA-loaded HepG2 cells, demonstrating their LDAI/oxLDAI activity. Statistical significance was assessed using one-way analysis of variance (ANOVA) with Tukey’s multiple comparison test against the untreated control, where **** *p* < 0.0001, *** *p* < 0.001, and. (**B-3**). Bioactive clove extracts (CE-MeOH and CE-CHCl_3_): % inhibition of DPPH by CE-MeOH and CE-CHCl_3_. (**B**) Antioxidant activity index values for CE. Graph showing the AAI values. Individual AAI values for each sample were calculated. The activity criteria based on the IC_50_ values were classified as very strong, strong, moderate, and weak. (**C**) Cells treated with CE-MeOH were visualized using phase-contrast and Oil Red O staining.

Neither CE-MeOH nor CE-CHCl_3_ exhibited cytotoxicity or lipotoxicity, as further confirmed by the experiments shown in Figure 2. This study specifically investigated the effects of LDAI and oxidized LDAI (oxLDAI) under nontoxic conditions, with specific evidence of the lack of lipotoxicity of linoleic acid (LA). Cytotoxicity was investigated using a CCK-8 assay and determination of CC_50_ and LC_50_ of CE-MeOH and CE-CHCl_3_. Previous studies have reported a direct proportional relationship between LA concentration and intracellular LD accumulation [10,13,14]. Based on these observations, CE-MeOH and CE-CHCl_3_ were treated simultaneously with 0.25 mM oleic acid (OA) and incubated for 24 h post-treatment. At a low concentration of 200 μg/mL, the CE-CHCl_3_ extract drastically decreased the number of LDs, as depicted in Figure 2. Previous studies have reported a directly proportional relationship between the LA concentration and intracellular LD accumulation [10,13,14]. Among the two tested extracts, only the CE-MeOH extract elicited a statistically significant reduction in LDs at 200 μg/mL of this extract. The corresponding LDAI values for CE-MeOH were 59.0%, 62.0%, and 94.0.0% (corresponding to different concentrations), which were significantly higher than those for the CE-CHCl_3_ sample.

### 3.2. Antioxidant Activity Index of Clove Extract and LD/oxLD Staining Experiment

**Figure 3:**
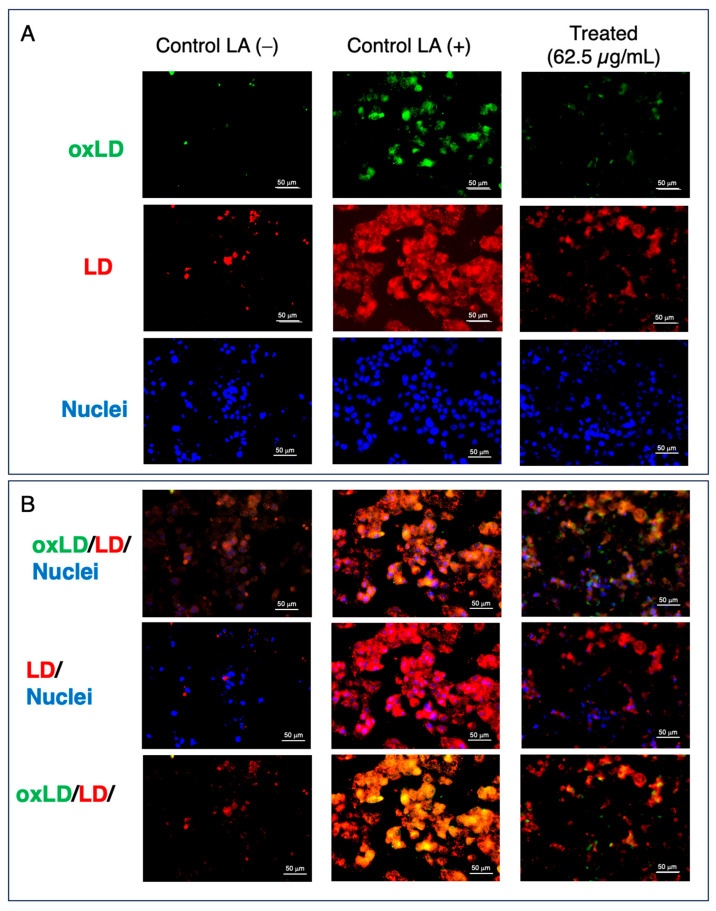
A. Visualization of cells treated with CE-MeOH using fluorescence staining techniques. (**A**) Oxidized LD, LD (red), and nucleus (blue) staining. (**B**) Combined oxidized (green), LD (red), and nucleus (blue) staining; combined LD (red) and nucleus (blue) staining; and combined oxidized (green) and LD (red) staining.

The antioxidant activity was determined using the DPPH assay, which classified extracts based on their AAI—very high with an IC_50_ ≥ 2.0, high with an IC_50_ between 1.0 and 2.0, moderate with an IC_50_ between 0.5 and 0.1, and low when below 0.5—as described in previous studies [13,14]. Accordingly, this study found that the CE-MeOH and CE-CHCl_3_ extracts showed considerable antioxidant activity, with AAI values of 1.21 and 0.70, respectively (Figure 2). The fluorescence staining techniques used to visualize LD/oxLD inhibition are shown in Figure 3.

### 3.3. Antioxidant Activity Index and LDAI of the Fractions of Clove Extract

#### 3.3.1. Antioxidant Activity Index of the Fractions of Clove Extract

Free radicals have become increasingly important in biological and medical research. These molecules, of which mitochondria are the major cellular source of ROS, are produced by various endogenous and exogenous processes in the body. An excess of free radicals can cause oxidative damage to critical biomacromolecules, such as proteins, lipids, and nucleic acids, leading to tissue injury in a wide range of degenerative and chronic diseases. This oxidative damage plays a role in the development of diseases and conditions such as atherosclerosis, aging, and cancer [16,17,18]. This study specifically investigated the AAI of clove extracts and two classic antioxidant compounds (Figure 2).

**Figure 4:**
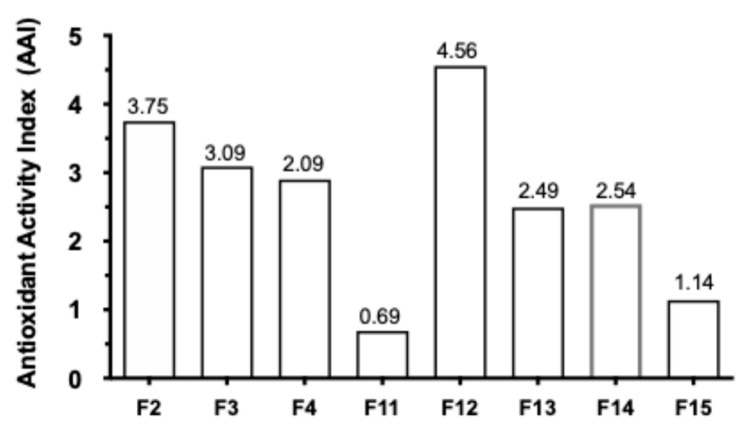
Bioactive clove CE fractions (IC_50_: % inhibition of DPPH). Antioxidant activity index values of CE fractions. Graph showing the AAI values. Individual AAI values for each sample were calculated. The activity criteria based on IC_50_ values were classified as very strong, strong, moderate, and weak.

Antioxidant activity was assayed using the DPPH assay, and the antioxidant activity index (AAI) was determined based on specific IC_50_ value criteria: IC_50_ ≥ 2.0, indicating very strong activity. This oxidative damage contributes to conditions such as atherosclerosis, aging, and cancer [16,17,18]. The results showed that the clove extract exhibited the highest antioxidant activity index. Chlorogenic acid (CA) and ascorbic acid (vitamin C, VC) are known antioxidants that display strong AAI values and are non-cytotoxic and lipotoxic to hepatocytes. However, a difference was observed in LDAI-related activity: only CA displayed moderate LDAI, whereas ascorbic acid did not show any significant inhibition; see Figure 4.

#### 3.3.2. LDAI of the Fractions of Clove Extract

The tested compounds demonstrated no cytotoxic or lipotoxic effects at concentrations below 50 µg/mL, establishing this concentration range as non-toxic for subsequent LDAI procedures. Under LA conditions, both F2 (12 µg/mL) and F12 (6 µg/mL) completely inhibited LDA/oxLDA (Table 1).

### 3.4. Metabolite Profiling, Dereplication of Secondary Metabolites from CE and Fractions, and Characterization of Potential Bioactive Compounds

#### 3.4.1. Metabolite Profiling and Dereplication of Secondary Metabolites from Clove Extract and Their Bioactive Fractions

The metabolomic analysis of bioactive clove extract emphasizes metabolite fingerprinting and quick dereplication of key compounds in complex mixtures of organic compounds, such as plant extracts. Liquid and gas chromatography linked to mass spectrometry (LC-MS and GC-MS) [16] and nuclear magnetic resonance (NMR) spectroscopy represent some of the analytical methods used for the direct identification of NPs in complex herbal matrices. Within the last ten years, automation techniques have been developed that make use of ^13^C-NMR in the dereplication of mixtures using a high-throughput approach that uses medium-field instruments (400 MHz), public-domain automation procedures, and dedicated software. Even though mass spectrometry is more sensitive, ^13^C-NMR and DEPT (135 and 90) are particularly effective for distinguishing diastereomers and resolving overlapping metabolites in ^1^H-NMR spectra.

**Figure 5:**
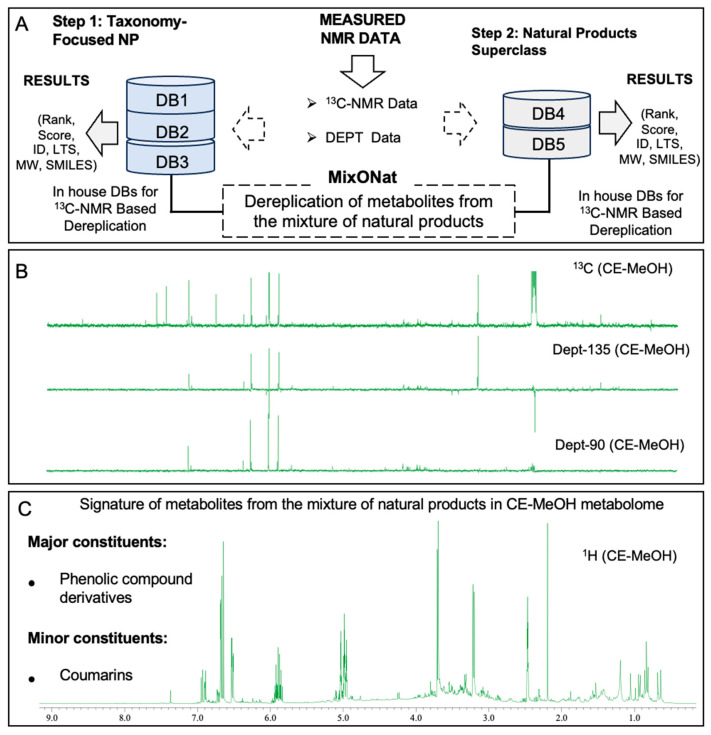
Fingerprinting and profiling of potential metabolites in bioactive CE food extract using 1D-NMR. (**A**) Overall operation of the MixONat program. This program matches the chemical shifts in the CE food extract experiment (^13^C, optionally DEPT-135, and DEPT-90) with those of the DB1-3 databases (Lamiaceae DB1 (1080 molecules), Myrtaceae DB2 (2020 molecules), and Euphorbiaceae experiments (6286 molecules), which contain molecular δC values selected from the LOTUS database. (**B**) ^13^C-NMR and DEPT (135 and 90) spectra of CE in DMSO, with *δ*_C_ ranging from 0 to 190 ppm. (**C**) ^1^H-NMR profile of the CE food extract in DMSO, with *δ*_H_ ranging from 0 to 12.0 ppm. The spectra were processed using JEOL software, and the chemical shift (*δ*) values are expressed in ppm.

This investigation utilized 1D-NMR techniques, such as ^1^H, ^13^C, and DEPT (135 and 90) NMR, for the profiling and rapid dereplication of potential chemicals within the raw clove extract, as shown in Figure 5. The ^1^H-NMR profiles and ^13^C-NMR metabolite dereplication gave indispensable information regarding the clove extract. The characteristic chemical shifts observed in the ^1^H-NMR spectra indicated the presence of aromatic rings within the essential oil of this plant. Metabolite fingerprinting and profiling using ^1^H-NMR showed signatures related to phenolic compounds.

##### Taxonomy-Focused NP Databases for ^13^C-NMR-Based Dereplication

Using LOTUS-DB, we built three databases: Lamiaceae-DB1, Myrtaceae-DB2, and Euphorbiaceae-DB-3 (Stored in Dibwe group internal database station). These databases can be used with MixONat for Taxonomy-Focused NP Databases for ^13^C-NMR-Based Dereplication, as shown in Figure 5. Myrtaceae and Lamiaceae represent two distinct plant families with remarkable metabolomic profiles and are reported to possess high concentrations of both phenolic compounds and essential oils. Myrtaceae fruits are also rich in sugars, organic acids, and vitamins, which enhance their nutritional and antioxidant capacities. Lamiaceae plants possess a broad variety of flavonoids and fatty acid derivatives with medicinal properties. Both families contain a variety of secondary metabolites; see Figure 5.

**Figure 6:**
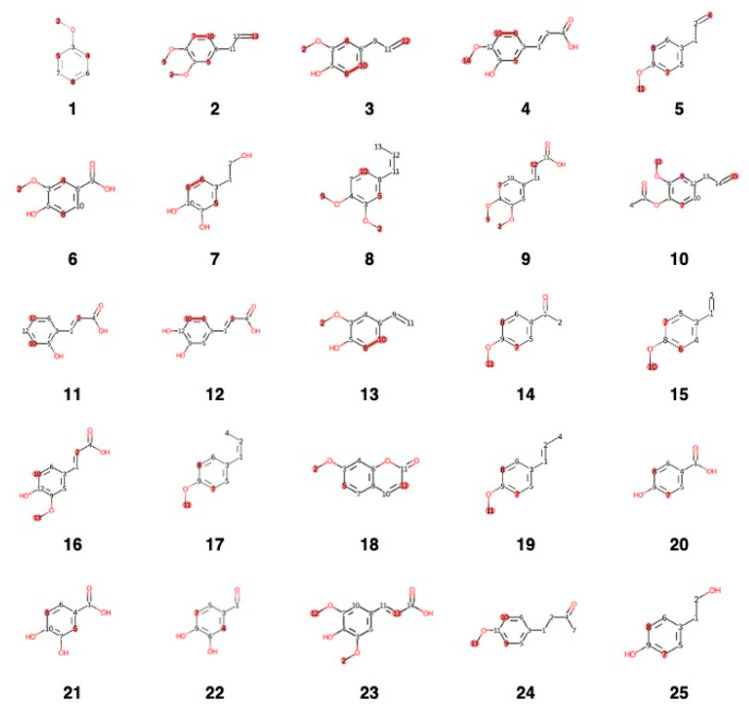
Top 25 phenolic compounds **1**–**25** (S**1**–S**25**) from MixONat using an internal database of natural products: rapid dereplication of HCA and HBA in CE (methanolic extract) using ^13^C-NMR and DEPT (135 and 90).

Dereplication from the Lamiaceae DB1 (ID: 980 molecules) revealed no molecules with perfect scores of 1.0. The top 10 compounds ranked 1 to 10, starting from 0.71 to 0.5, indicated that the missing carbon was mostly quaternary carbon with low intensities in the mixture extract, suggesting that these compounds were likely to be present in the extract. These molecules were identified as (**1**) rank: 1, ID: 276, 3-hydroxytyrosol (S**1**), CAS: CAS-10597-60-1, score: 0.75 (6/8 carbons); (**2**) rank: 2, ID: 416, O-methyleugenol (S**2**), CAS: CAS-93-15-2, score: 0.64 (7/11 carbons); (**3**) rank: 3, ID: 377, vanillic acid (S**3**), CAS: CAS-121-34-6, score: 0.62 (5/8 carbons); (**4**) rank: 4, ID: 353, p-eugenol (S**4**), CAS: CAS-97-53-0, score: 0.6 (6/10 carbons); (**5**) rank: 5, ID: 463, ferulic acid (S**5**), CAS: CAS-1135-24-6, score: 0.6 (6/10 carbons); (**6**) rank: 6, ID: 626, eugenol acetate (S**6**), CAS: CAS-93-28-7, score: 0.58 (7/12 carbons); (**7**) rank: 7, ID: 144, p-salicylic acid (S**7**), CAS: CAS-99-96-7, score: 0.57 (4/7 carbons), (**8**) rank: 8, ID: 275, gentisic acid (S**8**), CAS: CAS-490-79-9, score: 0.57 (4/7 carbons), (**9**) rank: 9, ID: 429, caffeic acid (S**9**), CAS: CAS-331-39-5, MW: 180.16, score: 0.56 (5/9 carbons), (**10**) rank: 10, ID: 70, 1,2,3-benzenetriol (S**10**), CAS: CAS-87-66-1, score: 0.5 (3/6 carbons) (Figure 6). From the Lamiaceae DB1 database (980 molecules), 10 out of 50 molecules were dereplicated, with scores ranging from 0.70 to 0.50 (see Figure 6; all structures are indicated in the Supplementary Materials).

Dereplication against the Myrtaceae DB2 (ID: 2217 molecules) (from MixONat DBs) identified no molecules with perfect scores of 1.0. The top 20 compounds ranked 1 to 20, starting from 0.71 to 0.5, indicated that the missing carbon was mostly quaternary carbon with low intensities in the mixture extract, suggesting that these compounds were likely to be present in the extract. These molecules were identified as rank: 1, ID: 1518: methoxyhydroquinone (S**51**), LTS0204713, score: 0.71 (5/7 carbons); rank: 2, ID: 1861: 4-dihydroxybenzoic acid (S**52**), LTS0018765, score: 0.71 (5/7 carbons); rank: 3, ID: 524: Eugenol (S**53**), LTS0052342, Score: 0.7 (7/10 carbons); rank: 4, ID: 1024: methyl-3h-furan-2-carbaldehyde (S**54**), LTS0245708, score: 0.67 (4/6 carbons); rank: 5, ID: 610: bran oil (S**55**), LTS0143969, score: 0.6 (3/5 carbons); rank: 6, ID: 1652: guaiacol (S**56**), LTS0179228, score: 0.57 (4/7 carbons); rank: 7, ID: 435: methyl anis (S**57**), LTS0113372, score: 0.56 (5/9 carbons); rank: 8, ID: 763: 4-dihydroxycinnamic acid (S**58**), LTS0128050, score: 0.56 (5/9 carbons); rank: 9, ID: 377: eremophilene (S**59**), LTS0101219, score: 0.53 (8/15 carbons); rank: 10, ID: 1748: methyl 3,4-dihydroxybenzo (S**60**), LTS0057841, score: 0.5 (4/8 carbons); rank: 11, ID: 530: ethyl anis (S**61**), LTS0184041, score: 0.5 (5/10 carbons); rank: 12, ID: 1191: tarragon (S**62**), LTS0245226, score: 0.5 (5/10 carbons); rank: 13, ID: 2133: vanillic acid (S**63**), LTS0229113, score: 0.5 (4/8 carbons); rank: 14, ID: 1967: chavibetol (S**64**), LTS0244260; score: 0.5 (5/10 carbons); rank: 15, ID: 990: -chloro-1-(4-hydroxy-3-methoxyphenyl)propane-1,2-diol (S**65**), LTS0132524, score: 0.5 (5/10 carbons); rank: 16, ID: 1324: compound name (S**66**), LTS0086128, score: 0.5 (5/10 carbons); rank: 17, ID: 2078 isoeugenol (S**67**), LTS0136836, score: 0.5 (5/10 carbons); rank: 18, ID: 866: (2r)-3-(4-hydroxy-3-methoxyphenyl)propane-1,2-diol (S**68**), LTS0114565, score: 0.5 (5/10 carbons); rank: 19, ID: 1882: -(4-hydroxy-3-methoxyphenyl)propane-1,2-diol (S**69**), LTS0016014, score: 0.5 (5/10 carbons); rank: 20, ID: 699: eugenyl acetate (S**70**), LTS0165886, MW: 206.24, score: 0.5 (6/12 carbons). Dereplication from the Myrtaceae DB2 database, which contains 2217 molecules, yielded 20 molecules among 50 molecules dereplicated with scores ranging from 0.70 to 0.50 (all structures are indicated in the Supplementary Materials).

Dereplication from Euphorbiaceae DB3 (ID: 6286 molecules) revealed no molecules with perfect scores of 1.0. The top 10 compounds ranked 1 to 20, starting from 0.71 to 0.5, indicating that the missing carbon was mostly quaternary carbon with low intensities in the mixture extract, suggesting that these compounds were likely to be present in the extract. These molecules were identified as rank: 1, ID: 5339: a pyridine alkaloid with Q182990 (S**101**), LTS0008205, score: 0.75 (3/4 carbons); rank: 2, ID: methoxyhydroquinone (S**102**), LTS0204713, score: 0.71 (5/7 carbons); rank: 3, ID: 2781: a pyridine alkaloid with Q725173892 (S**103**), LTS0182439, score: 0.71 (5/7 carbons); rank: 4, ID: 5295: 4-dihydroxybenzoic acid (S**104**), LTS0018765, score: 0.71 (5/7 carbons); rank: 5, ID: 1489: eugenol (S**105**), LTS0052342, score: 0.7 (7/10 carbons); rank: 6, ID: 726: p-anisic acid (S**106**), LTS0123492, score: 0.62 (5/8 carbons); rank: 7, ID: 2656: with Q831074201 (S**107**), LTS0173819, score: 0.57 (4/7 carbons); rank: 9, ID: 1922: with Q1044004643 (S**109**), LTS0144355, score: 0.57 (4/7 carbons); rank: 10, ID: 2174: 4-dihydroxycinnamic acid (S**110**), LTS0128050, score: 0.56 (5/9 carbons). Dereplication from Euphorbiaceae DB3 (6286 molecules) identified 10 molecules among 50 molecules dereplicated with scores ranging from 0.70 to 0.50 (all structures are indicated in the Supplementary Materials).

#### 3.4.2. Identification and Characterization of Potential Bioactive Lipid Droplet Accumulation Inhibitor and Antioxidants from Clove Fractions

Extensive NMR analysis of the compounds isolated from clove bioactive fractions led to the identification of potential bioactive agents targeting lipid droplet accumulation inhibitors and antioxidants.

The bioactive fraction F2 was subsequently processed for dereplication using DB1-3 Taxonomy-Focused NP Databases for ^13^C-NMR-Based Dereplication, leading to the identification of several chemical constituents. Constituents with scores in the range of 1 to 0.7 from each DB were selected and are listed as follows from DB1: rank: 1, ID: 626, p-eugenolugenolO-methyleugenol (S**151**), CAS: CAS-9methylferulic206.24, Scop-salicylic2/12 carbonprotocatechuic ID: 353, Nagallic-Eugenol (**2**ferulic), C isoferulic7-53-0, MW: 164.2, score: 1.0 (10/10 carbons), rank: 3, ID: 416, O-methyleugenol (S**1**), (**3**), CAS: CAS-93-15-2, MW: 178.23, score: 0.91 (10/11 carbons), rank: 4, ID: 276, name: 3-hydroxytyrosol (S**154**), (**4**), CAS: CAS-10597-60-1, MW: 154.16, score: 0.88 (7/8 carbons), rank: 5, ID: 631, methylferulic acid (S**155**), (**5**), CAS: CAS-2316-26-9, MW: 208.21, score: 0.82 (9/11 carbons), rank: 6, ID: 144, p-salicylic acid (S**156**), (**6**), CAS: CAS-99-96-7, MW: 138.12, score: 0.71 (5/7 carbons), rank: 7, ID: 274, protocatechuic acid (S**157**), CAS: CAS-99-50-3, MW: 154.12, score: 0.71 (5/7 carbons), rank: 8, ID: 387, gallic acid (S**158**), (**8**), CAS: CAS-149-91-7, MW: 170.12, score: 0.71 (5/7 carbons), rank: 9, ID: 463, ferulic acid (S**159**), (**9**), CAS: CAS-1135-24-6, MW: 194.18, score: 0.7 (7/10 carbons), rank: 10, ID: 462, isoferulic acid (S**160**), (**10**), CAS: CAS-537-73-5, MW: 194.18, score: 0.7 (7/10 carbons). All of the structures are described in detail in the Supplementary Materials.

Dereplication from Myrtaceae DB2 (6286 molecules) identified ten molecules with scores ranging from 0.70 to 0.99. Rank: 1, ID: 1967: chavibetol (S**171**), LTS0244260, MW: 164.2, score: 0.9 (9/10 carbons); rank: 2, ID: 2133: vanillic acid (S**172**), LTS0229113, MW: 168.15, score: 0.88 (7/8 carbons); rank: 3, ID: 1861: 4-dihydroxybenzoic acid (S**173**), LTS0018765, MW: 154.12, score: 0.86 (6/7 carbons), rank: 4, ID: 2000L: phenol (S**174**), LTS0092642, NMW: 94.11, score: 0.83 (5/6 carbons), rank: 5, ID: 524: eugenol (S**175**), LTS0052342, MW: 164.2, score: 0.8 (8/10 carbons), rank: 6, ID: 148: ferulic acid (S**176**), LTS0077328, MW: 194.18, score: 0.8 (8/10 carbons); rank: 7, ID: 763: 4-dihydroxycinnamic acid (S**177**), LTS0128050, MW: 180.16, score: 0.78 (7/9 carbons); rank: 8; ID: 56: -methoxybenzaldehyde (S**178**), LTS0124278; MW: 136.15; score: 0.75 (6/8 carbons); rank: 9; ID: 1748: methyl 3,4-dihydroxybenzo (S**179**), LTS0057841; MW: 168.15; score: 0.75 (6/8 carbons); rank: 10, ID: 1652: guaiacol (S**180**), LTS0179228, MW: 124.14, score: 0.71 (5/7 carbons) (Supplementary Materials). All of the structures are described in detail in the Supplementary Materials.

Furthermore, the bioactive fraction F12 was subsequently processed for dereplication using DB1-3 Taxonomy-Focused NP Databases for ^13^C-NMR-Based Dereplication, leading to the identification of the top ten constituents as follows: rank: 1, ID: 387, gallic acid (S**191**), CAS: CAS-149-91-7, MW: 170.12, score: 0.86 (6/7 carbons); rank: 2, ID: 316, p-Menth-3-en-8-ol (S**192**), CAS: CAS-18479-65-7, MW: 154.25, score: 0.8 (8/10 carbons); rank: 3, ID: 230, perillol (S**193**), CAS: CAS-536-59-4, MW: 152.23, score: 0.8 (8/10 carbons); rank: 4, ID: 70, 1,2,3-benzenetriol (S**194**), CAS: CAS-87-66-1, MW: 126.11, score: 0.67 (4/6 carbons); rank: 5, ID: 305, cis-beta-terpineol (S**195**), CAS: CAS-7299-40-3, MW: 154.25, score: 0.6 (6/10 carbons); rank: 6, ID: 402, 1-acetoxyoctane (S**196**), CAS: CAS-112-14-1, MW: 172.26, score: 0.6 (6/10 carbons); rank: 7, ID: 254, cis-limonene epoxide (S**197**), CAS: CAS-13837-75-7, MW: 152.23, score: 0.6 (6/10 carbons); rank: 8, ID: 384, (+)-2-hydroxypinocamphone (S**198**), CAS: CAS-24047-72-1, MW: 168.23, score: 0.6 (6/10 carbons); rank: 9, ID: 137, (+)-limonene (S**199**), CAS: CAS-5989-27-5, MW: 136.23, score: 0.6 (6/10 carbons), cumulated absolute difference: 2.89, rank: 10, ID: 109, p-Menta-1,3,8-triene (S**200**), CAS: CAS-18368-95-1, MW: 134.22, score: 0.6 (6/10 carbons). All of the structures are described in detail in Supplementary File S1.

Dereplication from Myrtaceae DB2 (6286 molecules) identified ten molecules with scores ranging from 0.70 to 0.99. rank: 1, ID: 1266: Galop (S211), LTS0222857, score: 0.86 (6/7 carbons); rank: 2, ID: 1715: ethyl gall (S212), LTS0270645, score: 0.78 (7/9 carbons); rank: 3, ID: 16: ethyl acetate (S213), LTS0196824, score: 0.75 (3/4 carbons); rank: 4, ID: 1134: -nonanol (S214), LTS0264829, Score: 0.67 (6/9 carbons); rank: 5, ID: 778: nonan-1-ol (S215), LTS0157379, score: 0.67 (6/9 carbons); rank: 6, ID: 1664: (2s)-nonan-2-ol (S216), LTS0186068, score: 0.67 (6/9 carbons); rank: 7, ID: 1966: -nonanone (S217), LTS0245014, score: 0.67 (6/9 carbons); rank: 8, ID: 463: (+)-2-undecanol (S218), LTS0195028, score: 0.64 (7/11 carbons); rank: 9, ID: 813: -undecanol (S219), LTS0141928, score: 0.64 (7/11 carbons); rank: 10, ID: 1952: caprylic acid (S220), LTS0254176, score: 0.62 (5/8 carbons) (Supplementary Materials). All structures are described in detail in the Supplementary Materials.

Twenty constituents were selected from the identified and dereplicated compounds of F2 and F12. Ten molecules were identified in F2: eugenol acetate (PC**1**), p-eugenol (PC**2**), O-methyleugenol (PC**3**), 3-hydroxytyrosol (PC**4**), methylferulic acid (PC**5**), p-salicylic acid (PC**6**), protocatechuic acid (PC**7**), gallic acid (PC**8**), ferulic acid (PC**9**), and isoferulic acid (PC**10**). In fr12, ten molecules, gallic acid (PC**11**), p-Menth-3-en-8-ol (PC**12**), perillol (PC**13**).; benzenetriol, (PC**14**), cis-beta-terpineol, (PC**15**), 1-acetoxyoctane (PC**16**), cis-limonene epoxide (PC**17**), (+)-2-hydroxypinocamphone (PC**18**), (+)-limonene (PC**19**), and p-Menta-1,3,8-triene (PC**20**) were targeted for further LDAI/oxLDAI investigation under LA. All of the structures are described in detail in the Supplementary Materials.

##### Natural Products Superclass Databases for ^13^C-NMR-Based Dereplication

Further, the dereplication process was extended to newly built in-house NP Superclass DBs to identify metabolites not previously reported in DB1–3. The dereplication process for DB-4 and DB-5 revealed several small peptides and carbohydrates, respectively, that were not reported in DB1–3. DB-4 comprises 1913 small peptide molecules identified as minor metabolite components of the DB. Both F2 and F12 were first suggested to be rapidly dereplicated from the Natural Products Superclass DB-4.

**Figure 7:**
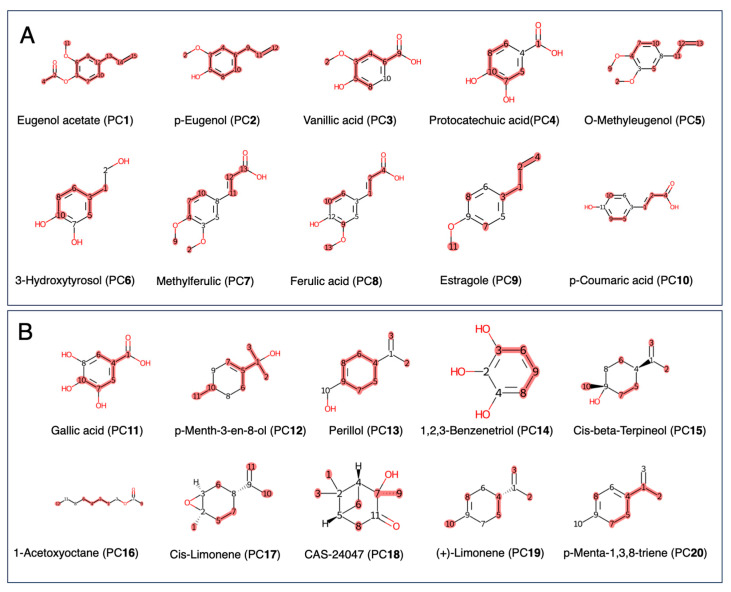
The dereplication of metabolites in the F2 and F12 mixtures was performed using an internal database of natural product molecules via MixONat. (**A**) Top ten compounds from ^13^C-NMR fast dereplication in F2. (**B**) Top ten molecules from the ^13^C-NMR fast dereplication in F12.

From F2, dereplication from small peptide DB-4 (ID: 2685 molecules) identified the top 10 additional molecules with scores ranging from 1.0 to 0.75. Rank: 1, ID: 1250: aminophenol (S**231**), LTS0118167, MW: 109.13, score: 1.0 (6/6 carbons); rank: 2, ID: 766: amino-3-(1 h-imidazol-2-yl)propanoic acid (S**232**), LTS0059060, MW: 155.15, score: 0.83 (5/6 carbons); rank: 3, ID: 1059: Dl-dencichin (S**233**), LTS0142657, MW: 176.13, score: 0.8 (4/5 carbons); rank: 4, ID: 1265 with Q409824 (S**234**), LTS0085766, MW: 176.13, score: 0.8 (4/5 carbons); rank: 5, ID: 1431: amino-4-chloropent-4-enoic acid (S**235**), LTS0156857, MW: 149.58, score: 0.8 (4/5 carbons); rank: 6, ID: 934: S-methyl-thio-cysteine (S**236**), LTS0041146, MW167.25, score: 0.75 (3/4 carbons); rank: 7, ID: 1912: amino-3-(5-hydroxypyridin-2-yl)propanoic acid (S**237**), LTS0150940, MW: 182.18, score: 0.75 (6/8 carbons); rank: 8, ID: 1320: (2s)-2-(hydroxyamino)butanedioic acid (S**238**), LTS0268578, MW: 149.1, score: 0.75 (3/4 carbons); rank: 9, ID: 535: -methyl-2h-pyridine-1,3-dicarboxylic acid (S**239**), LTS0045712, MW: 183.16, score: 0.75 (6/8 carbons); rank: 10, ID: 526: -(methylamino)benzamide (S**240**), LTS0239756, MW: 150.18, score: 0.75 (6/8 carbons) (Figure 7).

From F12, dereplication from the small peptide DB-4 (ID: 2685 molecules) identified the top 10 additional molecules with scores ranging from 0.67 to 0.57. Rank: 1, ID: 726: norleucine (S**271**), LTS0132632, MW: 131.17, score: 0.67 (4/6 carbons); rank: 2, ID: 834: L-(−)-ornithine (S**272**), LTS0224949, MW: 132.16, score: 0.6 (3/5 carbons); rank: 3, ID: 1115: ornithine (S**273**), LTS0150033, MW: 132.16, score: 0.6 (3/5 carbons); rank: 4 ID: 1400: L-ornithine (S**274**), LTS0093444, MW: 132.16, score: 0.6 (3/5 carbons), rank: 5, ID: 1246: hydroxynorvaline (S**275**), LTS0113357, MW: 133.15 score: 0.6 (3/5 carbons), cumulated absolute difference: 2.47; rank: 6, ID: 909: (2r)-3-amino-2-hydroxy-3-methylbutanoic acid (S**276**), LTS0006181, MW: 133.15, score: 0.6 (3/5 carbons); rank: 7, ID: 1632, LTS0004486, MW: 128.17, score: 0.57 (4/7 carbons), cumulated absolute difference: 1.12; cyclohexanecarboxylic acid (S**277**), rank: 8, ID: 1849: guanidinohexanoic acid (S**278**), LTS0047822, MW: 173.21, score: 0.57 (4/7 carbons); rank: 9, ID: 983: (2s)-2-amino-5-carbamimidamido-2-methylpentanoic acid (S**279**), LTS0034990, MW: 188.23, score: 0.57 (4/7 carbons); rank: 10, ID: 951: L-homoarginine (S**280**), LTS0176041, MW: 188.23, score: 0.57 (4/7 carbons) (Figure 7).

Our in-house-built Natural Product (NP) Superclass Database, specifically DB-5, which contains 2685 carbohydrate molecules, identified a significant class of minor metabolites. Both Fr2 and F12 were suggested to undergo rapid dereplication from the Natural Products Superclass DB-4. They indicated a low score of compounds for Fr2, while F12 indicated a score of compounds from 0.57 to 0.44.

F2: dereplication from carbohydrate DB-5 (ID: 2685 molecules) identified the top five molecules, with scores ranging from 1.0 to 0.5. Rank: 1, ID: 1195: Foscarnet (S**251**), LTS0038082, MW: 126.01, Score: 1.0 (1/1 carbons); rank: 2, ID: 2570: (1s,6r)-2-hydroxy-4-imino-5-oxo-7-oxabicyclo[4.1.0]hept-2-ene-3-carboxylic acid (S**252**), LTS0040576, MW: 183.12, score: 0.71 (5/7 carbons); rank: 3, ID: 1146: (1r,3s,4r,6s)-4,6-diaminocyclohexane-1,2,3-triol (S**253**), LTS0195613; MW: 162.19, score: 0.5 (3/6 carbons). Further, the dereplication process was extended to cover the newly developed rank: 4, ID: 1692:6-diaminocyclohexane-1,2,3-triol (S**254**), LTS0052927, MW: 162.19, score: 0.5 (3/6 carbons); rank: 5, ID: 128: denosine, 2′-deoxy- (S**255**), LTS0165339, MW: 251.24, score: 0.5 (5/10 carbons).

From F12, dereplication from carbohydrate DB-5 (ID: 2685 molecules) identified the top five additional molecules with scores ranging from 0.57 to 0.44. Rank: 1, ID: 2612, -bromo-4,5-dihydroxy-2-(hydroxymethyl)cyclohex-2-en-1-one (S**291**), LTS0033735, MW: 237.05, score: 0.57 (4/7 carbons); rank: 2, ID: 2595: (4r,5r,6r)-6-bromo-4,5-dihydroxy-2-(hydroxymethyl)cyclohex-2-en-1-one (S**292**), LTS0209560, MW: 237.05, score: 0.57 (4/7 carbons); rank: 3, ID: 2430: -(hydroxymethyl)cyclopent-4-ene-1,2,3-triol (S**293**), LTS0037932, MW: 146.14, score: 0.5 (3/6 carbons); rank: 4, ID: 1691: (1r,2s,3r,4s)-cyclohexane-1,2,3,4-tetrol (S**294**), LTS0064982, MW: 148.16, score: 0.5 (3/6 carbons); rank: 5, ID: 2021: (1s,5r,6r)-5,6-dihydroxy-3-(hydroxymethyl)-4-oxocyclohex-2-en-1-yl acetate (S**295**), LTS0017548, MW: 216.19, score: 0.44 (4/9 carbons).

## 4. Discussion

### 4.1. Clove Extract (CE) Inhibit LDAI Activity Under Linoeic Acid 

Neither of the tested extracts, CE-MeOH or CE-CHCl_3_, exhibited cytotoxicity or lipotoxicity. This investigation specifically focused on the effect of both LDAI and oxidized LDAI (oxLDAI) in conditions where neither extract was cytotoxic, and also emphasized that LA is non-lipotoxic. The cytotoxicity assays used were measured using the CCK-8 method. Figure 2 illustrates the marked decreases in intracellular LD formation by CE-CHCl_3_ at 200 μg/mL and CE-MeOH at the same concentration, yielding LDAI values of 92.5%, 93.5%, and 81.0%, respectively, which were higher than that of CE-CHCl_3_. Therefore, the CE-MeOH extract sample was further selected for an in-depth examination of its induced cellular morphological changes. Cells exposed to a CE-MeOH extract illustrated a distinct decrease in both size and overall number of intracellular lipid droplets relative to cells treated with lipids alone (Figure 2). Intracellular lipid droplets were stained with Oil Red O, a specific stain for lipid droplet accumulation, while cell nuclei were stained with Hoechst dye. The corresponding LDAI values of CE-MeOH were 92.5%, 93.5%, and 81.0%, demonstrating values higher than those for the CE-CHCl3 extract. At 250 µg/mL, a remarkably dose-dependent inhibitory effect was observed. The phenomenon of a decreased number and smaller size of the droplets was evident, which exceeded those noted at a concentration of 125 µg/mL. These results suggest that CE-MeOH significantly inhibits lipid droplet formation in a dose-dependent manner in the cellular uptake of fatty acid in cells (Figure 2).

### 4.2. The Antioxidant Activity Index of Clove Extract and LD/oxLD Staining Experiment

This study showed that the CE-MeOH and CE-CHCl_3_ extracts possessed potent antioxidant activity with AAI values of 1.21 and 0.70, respectively. Based on previous reports that LDAIs and antioxidants in plant nutraceuticals and functional foods alone or in combination may potentially manage metabolic disorders, the two above-mentioned extracts were further assayed for LDAI activity on HepG2 cells loaded with LA. Our previous study revealed that LDA and its oxidized form are formed under LA conditions, mediated by TAG build-up and oxTAG[TAG-(OOH)n = 3]. In addition, dietary extracts and their bioactive components induced LDAI and oxLDAI. In this study, CE-MeOH elicited an LDAI/oxLDAI response at 62.5, 125, and 250 μg/mL. Both CE-MeOH and CE-CHCl_3_ showed direct antioxidant activity (Figure 2 and Figure 3). As illustrated in Figure 3, in the imaging experiment conducted under LA-loaded conditions, LDA was decreased in the treated group under phase contrast. Fluorescence imaging after the phase contrast revealed an increase in red-stained LD and green-stained oxLD in the LA-loaded group [19]. In contrast, treatment with CE-MeOH resulted in a reduction in the accumulation of red LD and green oxLD in cells loaded with LA (Figure 3).

### 4.3. Evaluation of Antioxidant Effect and LDAI Activity of Fractions from Clove Extract

#### 4.3.1. Antioxidant Activity Index of Fractions of Clove Extract

The antioxidant activity index (AAI) of clove extracts was assessed against known antioxidants, including chlorogenic acid (CA) and ascorbic acid (vitamin C, VC). The results showed that clove extract attained the highest AAI, with CA giving a moderate LDAI and ascorbic acid demonstrating no effective inhibition. This was in good agreement with the fact that not all direct antioxidants can block LDA/oxLDA through a mechanism that could be linked to Keap1-Nrf2 pathway modulation. In addition to its high AAI value, the clove extract also exhibited high values of LDAI. Based on this study, a full lipidomic analysis is recommended to investigate the changes in intracellular neutral lipids, particularly triacylglycerols (TAGs) and their oxidized molecular species, in response to LA treatment. Additional data are represented in Figure 4 by the values of the antioxidant activity index for the bioactive extracts and provide individual AAI values for every sample and activity classes according to IC_50_.

Along with a strong AAI, clove extract expressed considerable LDAI. Fractions F2–F4 and F11–F14 displayed considerable antioxidant activities, most of them showing a higher capacity than the reference antioxidant deployed in the present study.

#### 4.3.2. LDAI of Fractions of Clove Extract

Since the compounds tested did not exhibit cytotoxic or lipotoxic effects below 50 µg/mL, this range was considered non-toxic under LDAI procedures. F2 and F12 completely inhibited LDA/oxLDA under LA conditions; therefore, they may also contain indirect antioxidant compounds interacting with antioxidant-related pathways such as Keap1-Nrf2. The two active fractions were then subjected together with its extract to a ^13^C-NMR-based dereplication using the ^13^C-NMR-based dereplication.

### 4.4. Metabolites Profiling; Dereplication of Secondary Metabolites from CE and Fractions; Characterization of Potential Bioactive Compounds

This study innovated and applied an approach to the metabolite profiling and dereplication method using Natural Products Superclass Databases for ^13^C-NMR-Based Dereplication and Taxonomy-Focused NP Databases for ^13^C-NMR-Based Dereplication. An appropriate database was created that overcame the limitations of existing natural product databases to allow for the direct dereplication of experimental data from mixtures of natural products. The newly developed databases that are compatible with MixONat allow for comparisons between reported and experimental ^13^C-NMR data analyses.

The ^13^C-NMR data from the extract confirmed the presence of phenolic compound molecules, including minor flavonoids and coumarins; compounds similar to eugenol, gallic acid, chlorogenic acid, and other phenolic derivative compounds; and flavonoid compounds such as quercetin and kaempferol. Secondary metabolites from clove extract and their bioactive fractions were profiled and dereplicated, NP Databases with a Taxonomy Focus for ^13^C-NMR Based Dereplication, using created three databases. Lamiaceae-DB1, Myrtaceae-DB2, and Euphorbiaceae-DB-3 extracted reported molecules from LOTUS-DB. MixONat for Taxonomy-Focused NP Databases for ^13^C-NMR-Based Dereplication were utilized with these databases. Two different plant families, Myrtaceae and Lamiaceae, are known to have large amounts of essential oils and phenolic chemicals, and they also have impressive metabolite profiles. The main phenolic compounds **1**–**25** (S**1**–S**25**) were identified from MixONat using internal natural product database, and this quick dereplication revealed HCA and HBA in CE (methanolic extract) as main constituents using DEPT and ^13^C-NMR (135 and 90). Previous studies indicate that Lamiaceae, Myrtaceae, and Euphorbiaceae species contain multiple components, with the main ones being phenolic compound derivatives. The top 50 compounds (S**1**–S**50**) were matched based on their ^13^C-NMR chemical shifts, DEPT (135 and 90) data, and intensities, which reflect the metabolite abundance. These 50 substances were ranked based on scores derived from comparisons with 7500 natural products from DB1–3 and their respective spectroscopic data. A variety of basic phenolic compound molecules, such as minor flavonoids and coumarins, were discovered through dereplication. Notably, the antioxidant chlorogenic acid (rank 28) and its derivatives (rank 39, 41, and 43) were identified in these samples. These findings suggest that clove extract (CE) is a potential resource for the development of functional foods and drug discovery targeting conditions such as MAFLD/MASH and other related diseases, as further detailed in the Supplementary Materials. Notably, the antioxidant chlorogenic acid (rank 28) and its derivatives (rank 39, 41, and 43) were identified in these samples. The natural product experimental data with the highest scores were compared with the existing literature data. An initial cohort of 50 compounds from each of the three distinct databases (DB1, DB2, and DB3) was identified. For DB1, these compounds correspond to ranks **1**–**50** and are denoted as S1–50. Similarly, DB2′s top 50 compounds are designated as ranks **1**–**50** or S**51**–**100**, and DB3′s top 50 compounds are identified as ranks **1**–**50** or S**101**–**150**. The basis for identifying these specific compounds rested on their characteristic ^13^C-NMR chemical shifts and measured intensities, both of which serve as critical indicators for estimating metabolite abundance. Following this identification process, the substances were systematically categorized. An initial cohort of 50 compounds from each of the three distinct databases (DB1, DB2, and DB3) was identified. This categorization was achieved through a direct comparative methodology, aligning them with the comprehensive collections of natural products housed within the specified databases of natural products cataloged in Lamiaceae DB1, Myrtaceae DB2, and Euphorbiaceae DB3. In addition, their classification also drew on detailed spectroscopic information contained in these databases.

In addition, to identify metabolites not previously reported in DB1–3, the dereplication process was extended to the newly built in-house NP Superclass DBs. The dereplication process for DB-4 and DB-5 revealed several small peptides and carbohydrates, respectively, which were not reported in DB1–3. DB-4, which comprises 1913 small peptide molecules identified as minor metabolite components. Both F2 and F12 were first suggested to be rapidly dereplicated from the Natural Products Superclass DB-4. F2: Dereplication from short peptide DB-4 (ID: 2685 molecules) revealed the top 10 extra molecules with scores ranging from 1.0 to 0.75 (Figure 7). F12: Dereplication from the short peptide DB-4 (ID: 2685 molecules) revealed the top 10 extra molecules with scores ranging from 0.67 to 0.57.

The in-house-built Natural Product (NP) Superclass Databases, specifically DB-5 with 2685 carbohydrate molecules, identified a significant class of minor metabolites. Both Fr2 and F12 were then proposed to rapidly undergo dereplication from the Natural Products Superclass DB-4. For Fr2, they had a low score of chemicals while F12 had a score of compounds between 0.57 and 0.44. F2: Dereplication from carbohydrate DB-5 (ID: 2685 molecules) identified five top-ranking molecules with scores ranging between 1.0 and 0.5. From F12: Dereplication from carbohydrate DB-5 (ID: 2685 molecules) identified the five top-ranking additional molecules with scores between 0.57 and 0.44.

Metabolomic analysis using 1D-NMR of the two bioactive extracts revealed a high concentration of hydroxybenzoic acids (HBAs) and hydroxycinnamic acids (HCAs) in F2 and hydroxybenzoic acids (HBAs) and hydroxycinnamic acids (HCAs) in F12 (Figure 7), which are likely responsible for the observed LDAI activity. Further investigation into the specific chemicals is necessary to identify the compounds that demonstrate strong AAI and LDAI/oxLDAI, as well as to determine the synergistic effects of this extract. Additionally, it is important to understand the key mechanism of action under LA conditions targeting the lipidomic analysis and gene expression of antioxidant enzyme; lipid metabolism; antioxidant enzyme; mitochondrial biogenesis; β-oxidation; and cardiolipin remodeling.

### 4.5. Strengths, Limitations, and Future Prospects

An important strength of the present work is the fact that the clove methanolic extract significantly suppressed both LDA and oxLDA formation in LA-induced HepG2 cells, importantly without cytotoxicity. CE-MeOH exhibited a high AAI value (1.21), which suggests the promising dual role of the sample as an antioxidant and a potent lipid droplet accumulation inhibitor. Metabolomic analysis using 1D-NMR enables rapid compound dereplication and indicated that the extract is rich in hydroxybenzoic acids (HBAs) and hydroxycinnamic acids (HCAs), which may be considered responsible for the observed LDAI activity. In addition, the F2 and F12 fractions showed high activities, achieving complete LDA/oxLDA inhibition, and turned out to be a good source of HBAs and HCAs. These are very intriguing results that strongly place clove extract as a highly promising natural resource for developing therapeutic agents, functional foods, or nutraceuticals to manage MAFLD, MASH, and related metabolic disorders.

However, several limitations and challenges should be overcome in further studies. Since not even a strong antioxidant such as ascorbic acid showed significant LDAI activity, this indicates that the effect most likely involves an indirect mechanism and is not due to simple direct antioxidant action; it possibly involves the regulation of the Keap1-Nrf2 pathway. Thus, further research will be required to develop a more precise elucidation of the molecular mechanisms. Second, although 1D-NMR enables rapid compound dereplication, the resolution limits of the method mean that the complete identification of all constituents, including those with low-intensity quaternary carbons, remains challenging. Finally, comprehensive lipidomics analysis is critical for a full understanding of the inhibitory effect of CE-MeOH on changes in intracellular triacylglycerols and their oxidized molecular species, which are pivotal components in LA-induced hepatic steatosis.

Further studies should focus on such limitations and extend the investigations. The first priority will be the isolation and identification of indirect antioxidant compounds that are present in highly active fractions, F2 and F12, and extend the investigations to similar and different metabolites from diverse plant origins with potent LDAI, oxLDAI activity, followed by validation at the molecular level of their interactions with antioxidant-related pathways such as Keap1-Nrf2. Metabolome analysis of the extract and their bioactive fractions revealed the presence of various metabolites such as HBAs and HCAs as potential LDAI, oxLDAI candidates. The synergetic effect on such metabolites in extract level is needed to link it with the regulation of the gene expression such as antioxidant enzyme (*NQO1*, *HO1*, *GR*); lipid metabolism (*ATGL*, *DGAT1*, *SBEBP1*, *SCD1*, *FASN*); antioxidant enzyme (*SOD*, *GCLC*, *GCLM*, *CAT*); mitochondrial biogenesis (*PPARGC1A*, *NRF1*, *TFAM*, *PPARA*), β-oxidation (*CPT1A*), cardiolipin remodeling (*TAZ*, *CLS1*, *PNPLA8*, *LCLAT1*) together with our established oxidative lipidomic approach. This is expected to understand the importance in the prevention as well as to identify new drug targets and mechanisms of action against MAFLD/MASH from food origin. The second priority is to perform an exhaustive lipidomics study, which can provide scientific authentication of the therapeutic benefits of clove extract via the assessment of the fluctuation in TAG and its oxidized species, providing far better insight into the mechanistic actions. These crucial results are expected to pave the way for the development and clinical translation of clove extract-based active components into nutraceutical products for the prevention and management of both MAFLD and MASH in a safe and effective manner. The current study lays an important foundation for further in-depth nutraceutical studies and drug discovery.

## 5. Conclusions

This study found candidates that met the requirements for LDAI/oxLDAI as well as fractions with potent antioxidant qualities. The ability to quickly dereplicate compounds found in the CE food extract that were identified as similar HCAs and HBAs is a significant methodological accomplishment described in the study. The use of advanced ^13^C-NMR metabolomic analysis techniques enabled this. The collective results derived from these analyses strongly suggest that the identified active compounds, when considered in conjunction with their respective rich food extracts, represent exceptionally promising resources. These resources are posited to be valuable for ongoing efforts in the prevention and effective management of metabolic dysfunction-associated fatty liver disease (MAFLD) and its advanced inflammatory progression, known as metabolic dysfunction-associated steatohepatitis (MASH) [20,21,22,23,24,25,26,27,28,29,30,31,32,33,34,35,36,37,38,39,40,41,42]. Furthermore, the implications of these findings extend significantly into the realm of future scientific endeavors, indicating their potential utility and relevance for further comprehensive nutraceutical investigations and the critical process of drug discovery.

## Figures and Tables

**Table 1:** Antioxidant activity index and LDAI of the fractions of clove extract (CA: chlorogenic acid; VC: vitamin C).

Fraction	AAI	CC_50_	LC_50_	LDAI/oxLDAI (%)	
				12 μg/mL	25 μg/mL
F1	0	>250	>125	0	100
F2	3.75	>125	>125	35	100
F3	3.09	>125	>62.5	0	0
F4	2.09	>62.5	>62.5	0	0
F5	0	>250	>62.5	0	100
F6	0	>125	>62.5	0	0
F7	0	>125	>31.3	0	0
F8	0	>31.3	>31.3	0	49
F9	0	>15.6	>15.6	0	0
F10	0	>31.3	>31.3	100	100
F11	0.69	>125	>31.3	0	0
F12	4.56	>125	>62.5	100	100
F13	2.49	>250	>62.5	0	69
F14	2.54	>250	>125	0	0
F15	1.14	>250	>15.6	0	0
Control 1-CA	2.5	>250	>250	0	63
Control 2-VC	2.4	>250	>250	0	0

## Data Availability

Data are contained within the article and Supplementary Materials.

## Supplementary Materials

The following supporting information can be downloaded at https://www.mdpi.com/article/10.3390/metabo16010007/s1. File S1: Materials and Methods: 1. Instruments and chemicals. 2. Lipid droplet accumulation inhibition assay, 3. Metabolite profiling and fingerprinting via NMR analyses of clove extract and their fractions. Dereplication of bioactive methanolic extracts: Figure S1: Dereplication analysis from MixONat, structure of top 50 metabolites: compounds S**1**–S**10** from DB-1; Figure S2: Dereplication analysis from MixONat, structure of top 50 metabolites: compounds S**11**–S**20** from DB-1; Figure S3: Dereplication analysis from MixONat, structure of top 50 metabolites: compounds S**21**–S**30** from DB-1; Figure S4: Dereplication analysis from MixONat, structure of top 50 metabolites: compounds S**31**–S**40** from DB-1; Figure S5: Dereplication analysis from MixONat, structure of top 50 metabolites: compounds S**41**–S**50** from DB-1; Figure S6: Dereplication analysis from MixONat, structure of top 50 metabolites: compounds S**51**–S**60** from DB-2; Figure S7: Dereplication analysis from MixONat, structure of top 50 metabolites: compounds S**61**–S**70** from DB-2; Figure S8: Dereplication analysis from MixONat, structure of top 50 metabolites: compounds S**71**–S**80** from DB-2; Figure S9: Dereplication analysis from MixONat, structure of top 50 metabolites: compounds S**81**–S**90** from DB-2; Figure S10: Dereplication analysis from MixONat, structure of top 50 metabolites: compounds S**91**–S**100** from DB-2; Figure S11: Dereplication analysis from MixONat, structure of top 50 metabolites: compounds S**101**–S**110** from DB-3; Figure S12: Dereplication analysis from MixONat, structure of top 50 metabolites: compounds S**111**–S**120** from DB-3; Figure S13: Dereplication analysis from MixONat, structure of top 50 metabolites: compounds S**121**–S**130** from DB3; Figure S14: Dereplication analysis from MixONat, structure of top 50 metabolites: compounds S**131**–S**140** from DB-3; Figure S15: Dereplication analysis from MixONat, structure of top 50 metabolites: compounds S**141**–S**150** from DB-3. Step1: Dereplication of bioactive fractions: A. F2-D1 (20) S**151**–S**170**; Figure S16: Dereplication analysis of F2-D1 from MixONat, structure of top 20 metabolites: compounds S**151**–S**160** from DB1. Figure S17: Dereplication analysis of F2-D1 from MixONat: structure of top 20 metabolites, compounds S**161**–S**170** from DB1. Step1: Dereplication of bioactive fractions: B. F2-D2 (20) S**171**–S**190**; Figure S18: Dereplication analysis of F2-D2 from MixONat, structure of top 50 metabolites: compounds S**171**–S**180** from DB2. Figure S19: Dereplication analysis from MixONat, structure of the top 50 metabolites: compounds S**181**–S**190** from DB2. Step1: Dereplication of bioactive fractions: F12-D1 (20) S**191**–S**210**; Figure S20: Dereplication analysis of F12-D1 from MixONat, structure of the top 50 metabolites: compounds S**191**–S**200** from D1. Figure S21: Dereplication analysis from MixONat, structure of the top 50 metabolites: compounds S**201**–S**210** from D1. Step1: Dereplication of bioactive fractions: D. F12-D2 (20) S**211**–S**230**; Figure S22: Dereplication analysis of F12-D2 from MixONat, structure of the top 50 metabolites: compounds S**211**–S**220** from DB2. Figure S23: Dereplication analysis from MixONat, structure of the top 50 metabolites: compounds S**221**–S**230** from DB2. Step2: Dereplication of bioactive fractions: A. F2-D4 (20) S**231**–S**250**; Figure S24: Dereplication analysis of F2-DB4 from MixONat, structure of the top 50 metabolites: compounds S**231**–S**240** from DB-4. Figure S25: Dereplication analysis from MixONat; structure of the top 50 metabolites: compounds S**241**–S**250** from DB-4. Step2: Dereplication of bioactive fractions: B. F2-DB-5 (20) S**251**–S**270**; Figure S26. Dereplication analysis from MixONat, structure of top 50 metabolites: compounds S**251**–S**260** in DB-5. Figure S27. Dereplication analysis from MixONat, structure of top 50 metabolites: compounds S**261**–S**270** in DB-5. Step2: Dereplication of bioactive fractions: F12-DB-4 (20) S**271**–S**290**; Figure S28. Dereplication analysis from MixONat, structure of top 50 metabolites: compounds S**271**–S**280** in DB-4. Figure S29. Dereplication analysis from MixONat, structure of top 50 metabolites: compounds S**281**–S**290** in DB-4. Step2: Dereplication of bioactive fractions: D. F12-DB-5 (20) S**291**–S31**0**; Figure S30. Dereplication analysis from MixONat, structure of top 50 metabolites: compounds S**291**–S**300** in DB-5. Figure S31. Dereplication analysis from MixONat, structure of top 50 metabolites: compounds S**301**–S**310** in DB-5.

## Abbreviations

LDA, lipid droplet accumulation; LDAI, lipid droplet accumulation inhibition; MAFLD, metabolic dysfunction-associated fatty liver disease; MASH, metabolic dysfunction-associated steatohepatitis; oxLD, oxidized lipid droplet; OA, oleic acid; LA, linoleic acid; LD, lipid droplet; TAG, triacylglycerol; TAG-(OOH)n, triacylglycerol hydroperoxide; FFA, free fatty acid; LC/MS, liquid chromatography/mass spectrometry; NMR, nuclear magnetic resonance; LTS, lotus.
