# Supplementary material for: Antioxidant Clove Extract Inhibits Lipid Droplet Accumulation and Lipid Oxidation in Hepatocytes
Source: Metabolites. 2025 Dec 22;16(1):7. doi: 10.3390/metabo16010007 (PMC12843948; doi:10.3390/metabo16010007)
Supplement: Supplementary file 1 [file metabolites-16-00007-s001.zip › metabolites-4004383-supplementary.pdf]

Supporting Information

Antioxidant Clove Extract Inhibits Lipid Droplet Accumulation and Lipid Oxidation in Hepatocytes

Satomi Monde [1,†], Dya Fita Dibwe [2,†], Shion Iwasaki [1], and Shu-Ping Hui [2,*]

[1] Graduate School of Health Sciences, Hokkaido University, Kita-12, Nishi-5, Kita-Ku, Sapporo 060-0812, Japan;

[2] Faculty of Health Sciences, Hokkaido University, Kita-12, Nishi-5, Kita-Ku, Sapporo 060-0812, Japan;

* Correspondences: keino@hs.hokudai.ac.jp; Tel./Fax: +81-11-706-369

Supporting Information

Table of contents:

Materials and Methods P5
1. Chemicals and instruments P5
2. Lipid droplet accumulation inhibition assay. P5
3. Metabolite profiling and fingerprinting via NMR analyses of *Clove* extract and their fractions P6

Step1: Dereplication of Bioactive Methanolic Extract

Dereplication analysis from MixONat, structure of top 50 metabolites: compounds S**1**-S**50** in DB-1 P6

Figure S1. Dereplication analysis from MixONat, structure of top 50 metabolites: compounds S**1**–S**10** in DB-1 P7

Figure S2. Dereplication analysis from MixONat, structure of top 50 metabolites: compounds S**11**–S**20** in DB-1 P8

Figure S3. Dereplication analysis from MixONat, structure of top 50 metabolites: compounds S**21**–S**30** in DB-1 P9

Figure S4. Dereplication analysis from MixONat, structure of top 50 metabolites: compounds S**31**–S**40** in DB-1 P10

Figure S5. Dereplication analysis from MixONat, structure of top 50 metabolites: compounds S**41**–S**50** in DB-1 P11

Step1: Dereplication of Bioactive Methanolic Extract

Dereplication analysis from MixONat, structure of top 50 metabolites: compounds S**51**-S**100** in DB-2 P12

Figure S6. Dereplication analysis from MixONat, structure of top 50 metabolites: compounds S**51**–S**60** in DB-2 P13

Figure S7. Dereplication analysis from MixONat, structure of top 50 metabolites: compounds S**61**–S**70** in DB-2 P14

Figure S8. Dereplication analysis from MixONat, structure of top 50 metabolites: compounds S**71**–S**80** in DB-2 P15

Figure S9. Dereplication analysis from MixONat, structure of top 50 metabolites: compounds S**81**–S**90** in DB-2 P16

Figure S10. Dereplication analysis from MixONat, structure of top 50 metabolites: compounds S**91**–S**100** in DB-2 P17

Step1: Dereplication of Bioactive Methanolic extracts

Dereplication analysis from MixONat, structure of top 50 metabolites: compounds S**101**-S**150** in DB-3 P18

Figure S11. Dereplication analysis from MixONat, structure of top 50 metabolites: compounds S**101**–S**110** in DB-3 P19
Figure S12. Dereplication analysis from MixONat, structure of top 50 metabolites: compounds S**111**–S**120** in DB-3 P20
Figure S13. Dereplication analysis from MixONat, structure of top 50 metabolites: compounds S**121**–S**130** in DB-3 P21
Figure 14. Dereplication analysis from MixONat, structure of top 50 metabolites: compounds S**131**–S**140** in DB-3 P22
Figure S15. Dereplication analysis from MixONat, structure of top 50 metabolites: compounds S**141**–S**150** in DB-3 P23
Step1: Dereplication of bioactive fractions: A. F2-D1 (20) S**151**–S**170** P24
Figure S16: Dereplication analysis of F2-D1 from MixONat, structure of top 20 metabolites: compounds S**151**–S**160** from DB1 P25
Figure S17: Dereplication analysis of F2-D1 from MixONat, structure of top 20 metabolites: compounds S**161**–S**170** from DB1 P26
Step1: Dereplication of bioactive fractions: B. F2-D2 (20) S**171**–S**190** P27
Figure S18: Dereplication analysis of F2-D2 from MixONat, structure of top 50 metabolites: compounds S**171**–S**180** from DB2. P28
Figure S19: Dereplication analysis from MixONat, structure of the top 50 metabolites: compounds S**181**–S**190** from DB2. P29
Step1: Dereplication of bioactive fractions: C. F12-D1 (20) S**191**–S**210** P30
Figure S20: Dereplication analysis of F12-D1 from MixONat, structure of the top 50 metabolites: compounds S**191**–S**200** from D1. P31
Figure S21: Dereplication analysis from MixONat, structure of the top 50 metabolites: compounds S**201**–S**210** from D1. P32
Step1: Dereplication of bioactive fractions: C. F12-D2 (20) S**211**–S**230** P33
Figure S22: Dereplication analysis of F12-D2 from MixONat, structure of the top 50 metabolites: compounds S**211**–S**220** from DB2. P34
Figure S23: Dereplication analysis from MixONat, structure of the top 50 metabolites: compounds S**221**–S**230** from DB2. P35

Step2: Dereplication of bioactive fractions: A. F2-D4 (20) S**231**–S**250** P36

Figure S24: Dereplication analysis of F2-DB4 from MixONat, structure of the top 50 metabolites: compounds S**231**–S**240** from DB-4. P37

Figure S25: Dereplication analysis from MixONat; structure of top 50 metabolites: compounds S**241**–S**250** from DB-4. P38

Step2: Dereplication of bioactive fractions: B. F2-DB-5 (20) S**251**–S**270** P39

Figure S26. Dereplication analysis from MixONat, structure of top 50 metabolites: compounds S**251**–S**260** in DB-5 P40

Figure S27. Dereplication analysis from MixONat, structure of top 50 metabolites: compounds S**261**–S**270** in DB-5 P41

Step2: Dereplication of bioactive fractions: C. F12-DB-4 (20) S**271**–S**290** P42

Figure S28. Dereplication analysis from MixONat, structure of top 50 metabolites: compounds S**271**–S**280** in DB-4 P43

Figure S29. Dereplication analysis from MixONat, structure of top 50 metabolites: compounds S**281**–S**290** in DB-4 P44

Step2: Dereplication of bioactive fractions: D. F12-DB-5 (20) S**291**–S31**0** P45

Figure S30. Dereplication analysis from MixONat, structure of top 50 metabolites: compounds S**291**–S**300** in DB-5 P46

Figure S31. Dereplication analysis from MixONat, structure of top 50 metabolites: compounds S**301**–S**310** in DB-5 P47

Materials and Methods

1. *Chemicals and instruments*

General Experimental Procedures: NMR spectroscopy was carried out on a JEOL ECX400 Delta instrument with TMS serving as the internal standard; chemical shifts were recorded as δ values. HR-ESI-MS analysis utilized an LTQ Orbitrap XL device (Thermo Fisher Scientific Inc., San Jose, CA, USA). Methanol was sourced from Wako. Cell culture materials, such as high-glucose DMEM, DPBS, trypsin EDTA, FBS, and penicillin-streptomycin (100 U/mL), were obtained from Gibco (Life Technologies, Carlsbad, CA, USA). Additional cell culture supplies were acquired from Corning (NY, USA). NMR data were gathered using a 400 MHz JNM-ECX400P instrument (JOEL, Japan). Spectral processing was conducted with JOEL software, and chemical shifts (δ) were reported in ppm. OA was purchased from Cayman Chemical (Ann Arbor, MI, USA), and absorbance was measured using ARVO-MX (Perkin Elmer, Waltham, MA, USA) according to previously described methods

2. *Lipid droplet accumulation inhibition assay*

The activity of LDAI was evaluated using an Oil Red O assay conducted in 24-well plates (n = 4 per treatment), focusing on the staining of lipid droplets (LDs) in cultured hepatocytes, as per the manufacturer's guidelines. HepG2 cells, at a density of 1.5×10^4 per well, were supplemented with 10% FBS, cultured, and then seeded into 35 mm dishes. After 24 hours, they were treated with the test samples. Oil Red O, a dye that dissolves in fat, is commonly employed to stain neutral lipids in LDs, as previously documented. Subsequently, the inhibition of LDs was quantified for the test BEs by comparing it to the untreated control group (+OA) and normalizing the LDA absorbance values (%), as previously reported. The LD and oxLDs

staining assay was conducted as previously described, with some modifications combining phasecontrast and fluorescence images.

3. Metabolite profiling and fingerprinting via NMR analyses of *Clove* extract and fractions

Typically, natural products are produced as a combination of structurally related compounds rather than individual substances. Dereplication of bioactive methanolic extract and their factions are presented below using our step1 and step2 strategies:

Step1: Dereplication of Bioactive Methanolic Extract

Dereplication analysis from MixONat, structure of top 50 metabolites: compounds S**1**-S**50** in DB-1

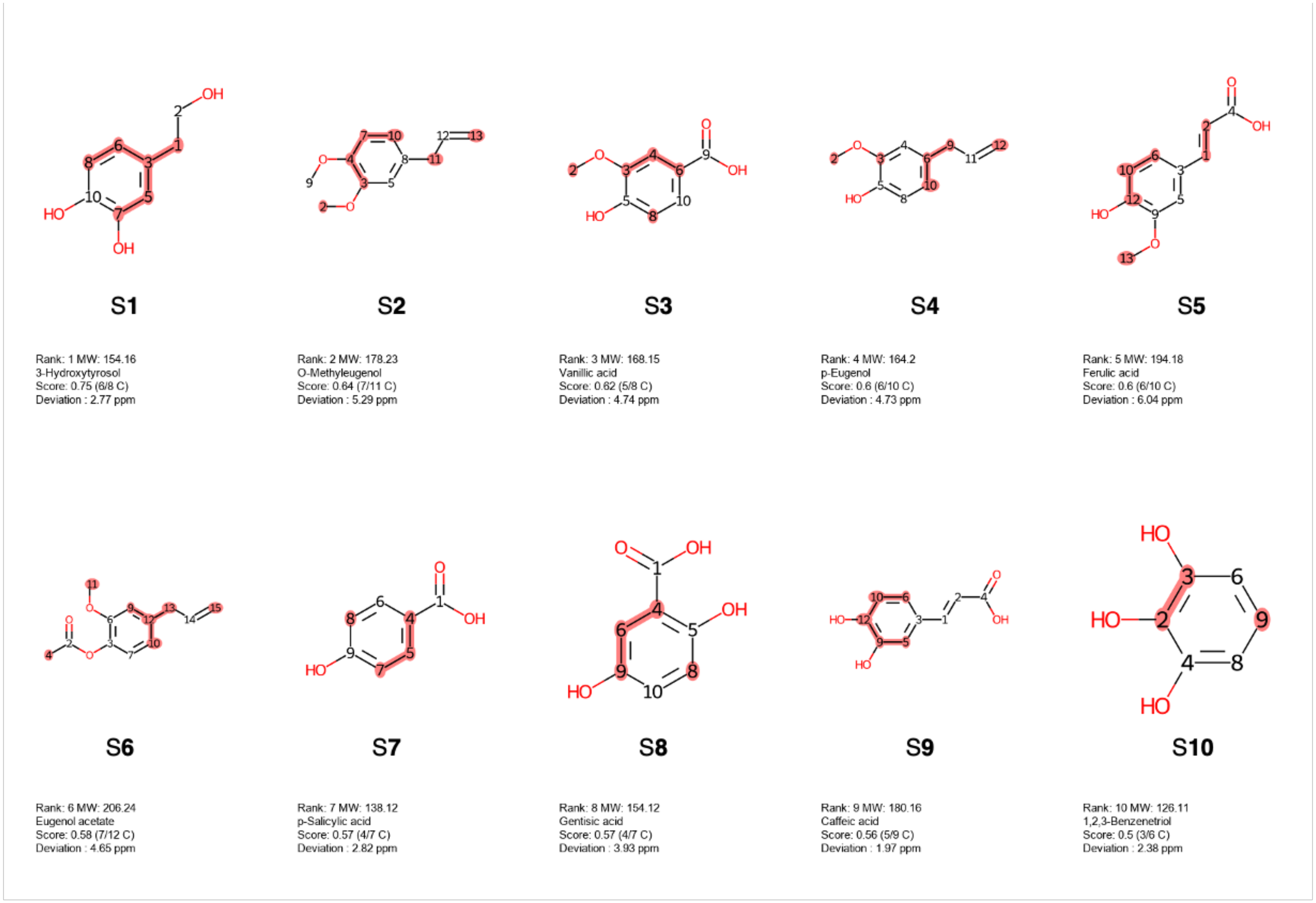

Figure S1. Dereplication analysis from MixONat, structure of top 50 metabolites: compounds S**1**–S**10** in DB-1

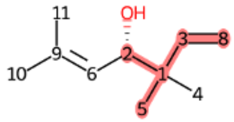

S11

Rank: 11 MW: 154.25
(-)-Artemisia alcohol
Score: 0.5 (5/10 C)
Deviation : 2.84 ppm

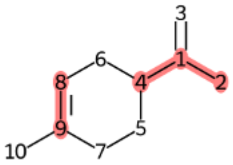

S12

Rank: 12 MW: 136.23
Limonene
Score: 0.5 (5/10 C)
Deviation : 3.41 ppm

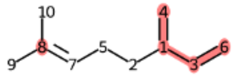

S13

Rank: 13 MW: 136.23
beta-Myrcene
Score: 0.5 (5/10 C)
Deviation : 3.45 ppm

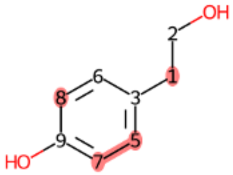

S14

Rank: 14 MW: 138.16
Tyrosol
Score: 0.5 (4/8 C)
Deviation : 3.62 ppm

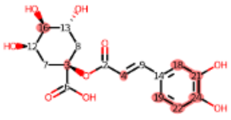

S15

Rank: 15 MW: 354.31
CAS-1241-87-8
Score: 0.5 (8/16 C)
Deviation : 5.53 ppm

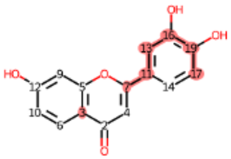

S16

Rank: 16 MW: 270.24
5-Deoxyluteolin
Score: 0.47 (7/15 C)
Deviation : 3.44 ppm

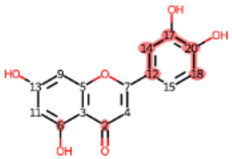

S17

Rank: 17 MW: 286.24
Luteolin
Score: 0.47 (7/15 C)
Deviation : 3.83 ppm

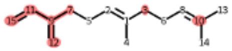

S18

Rank: 18 MW: 204.35
(E)-beta-Farnesene
Score: 0.47 (7/15 C)
Deviation : 4.93 ppm

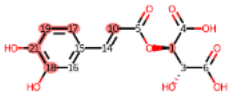

S19

Rank: 19 MW: 312.23
trans-Caftaric acid
Score: 0.46 (6/13 C)
Deviation : 4.66 ppm

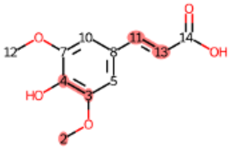

S20

Rank: 20 MW: 224.21
Sinapic acid
Score: 0.45 (5/11 C)
Deviation : 2.96 ppm

Figure S2. Dereplication analysis from MixONat, structure of top 50 metabolites: compounds S**11**–S**20** in DB-1

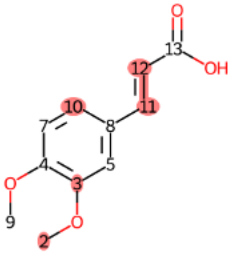

S21

Rank: 21 MW: 208.21
Methylferulic acid
Score: 0.45 (5/11 C)
Deviation : 3.12 ppm

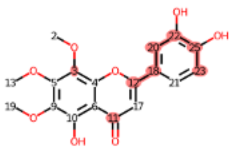

S22

Rank: 22 MW: 360.31
Sideritiflavone
Score: 0.44 (8/18 C)
Deviation : 3.29 ppm

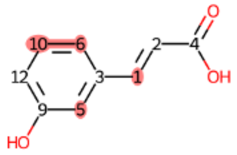

S23

Rank: 23 MW: 164.16
m-Coumaric acid
Score: 0.44 (4/9 C)
Deviation : 4.21 ppm

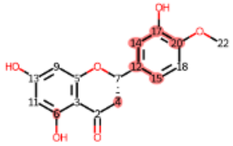

S24

Rank: 24 MW: 302.28
(-)-Hesperetin
Score: 0.44 (7/16 C)
Deviation : 3.26 ppm

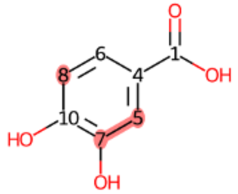

S25

Rank: 25 MW: 154.12
Protocatechuic acid
Score: 0.43 (3/7 C)
Deviation : 1.34 ppm

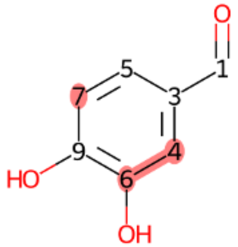

S26

Rank: 26 MW: 138.12
Catechaldehyde
Score: 0.43 (3/7 C)
Deviation : 2.49 ppm

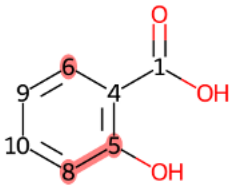

S27

Rank: 27 MW: 138.12
Salicylic acid
Score: 0.43 (3/7 C)
Deviation : 3.33 ppm

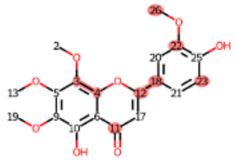

S28

Rank: 28 MW: 374.34
7-O-Methylsudachietin
Score: 0.42 (8/19 C)
Deviation : 4.08 ppm

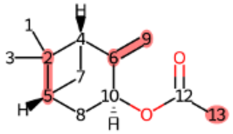

S29

Rank: 29 MW: 194.27
CAS-1686-15-3
Score: 0.42 (5/12 C)
Deviation : 2.62 ppm

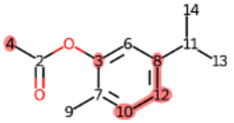

S30

Rank: 30 MW: 192.25
Carvacryl acetate
Score: 0.42 (5/12 C)
Deviation : 2.93 ppm

Figure S3. Dereplication analysis from MixONat, structure of top 50 metabolites: compounds S**21**–S**30** in DB-1

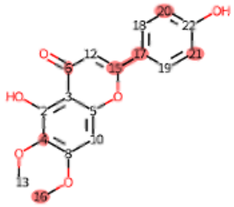

S31

Rank: 31 MW: 314.29
Circimaritin
Score: 0.41 (7/17 C)
Deviation : 4.78 ppm

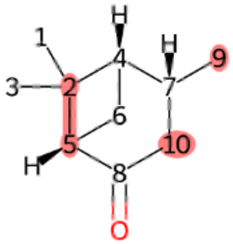

S32

Rank: 32 MW: 152.23
cis-Verbanone
Score: 0.4 (4/10 C)
Deviation : 0.6 ppm

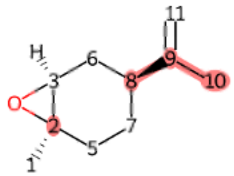

S33

Rank: 33 MW: 152.23
trans-Limonene epoxide
Score: 0.4 (4/10 C)
Deviation : 0.66 ppm

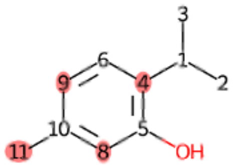

S34

Rank: 34 MW: 150.22
m-Thymol
Score: 0.4 (4/10 C)
Deviation : 1.26 ppm

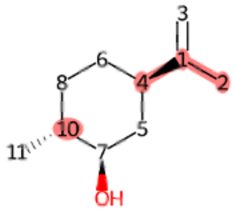

S35

Rank: 35 MW: 154.25
Dihydrocarveol
Score: 0.4 (4/10 C)
Deviation : 1.48 ppm

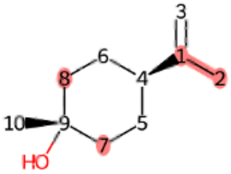

S36

Rank: 36 MW: 154.25
cis-beta-Terpineol
Score: 0.4 (4/10 C)
Deviation : 1.51 ppm

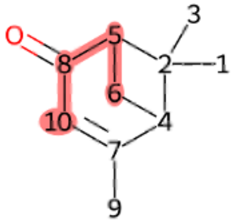

S37

Rank: 37 MW: 150.22
Verbenone
Score: 0.4 (4/10 C)
Deviation : 1.58 ppm

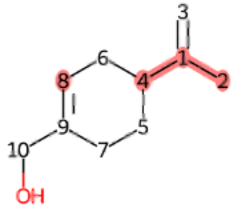

S38

Rank: 38 MW: 152.23
Perillol
Score: 0.4 (4/10 C)
Deviation : 1.6 ppm

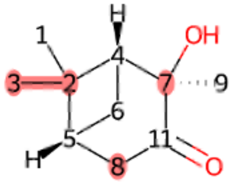

S39

Rank: 39 MW: 168.23
CAS-24047-72-1
Score: 0.4 (4/10 C)
Deviation : 1.7 ppm

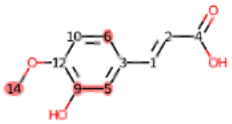

S40

Rank: 40 MW: 194.18
Isoferulic acid
Score: 0.4 (4/10 C)
Deviation : 1.75 ppm

Figure S4. Dereplication analysis from MixONat, structure of top 50 metabolites: compounds S**31**–S**40** in DB-1

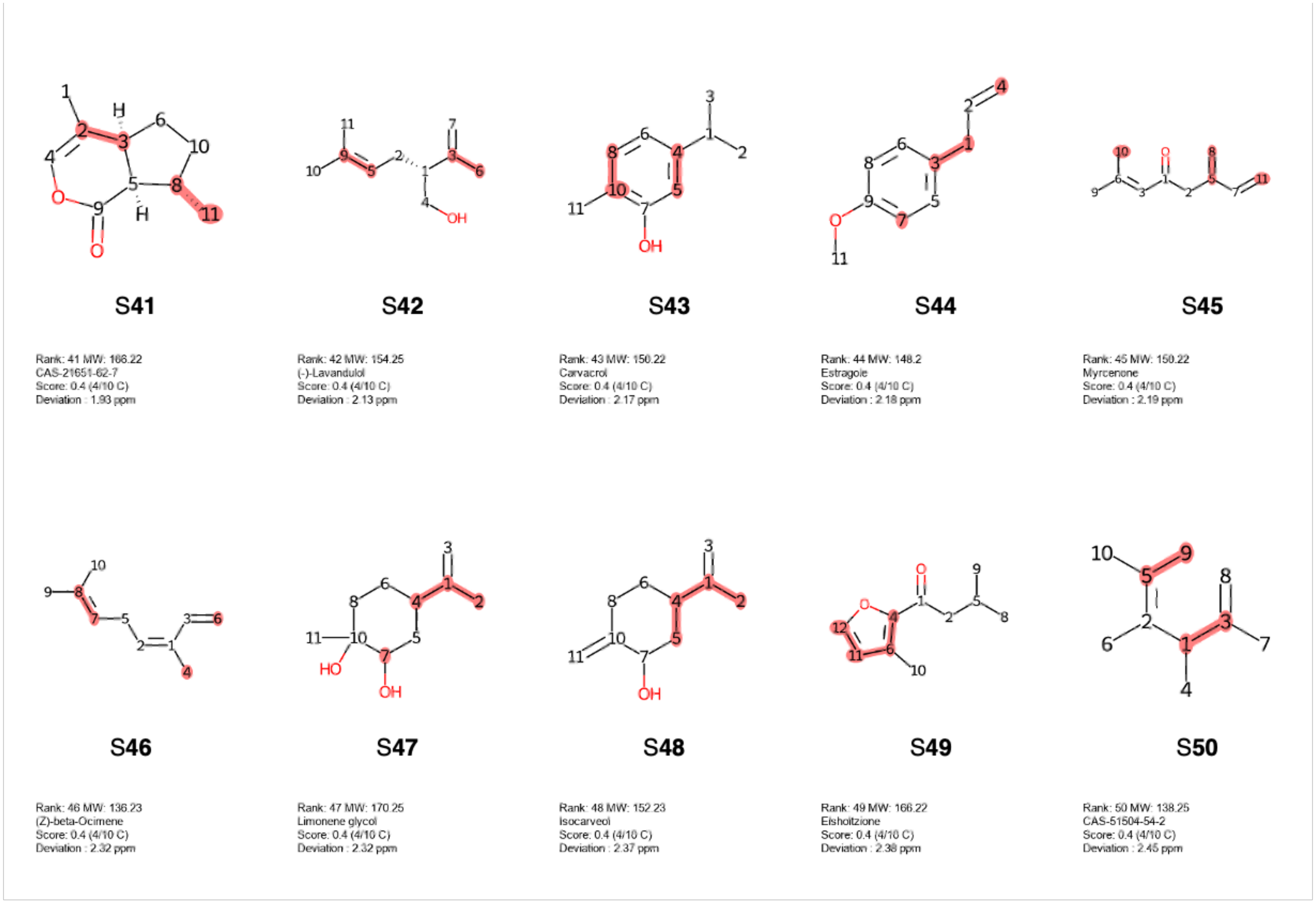

Figure S5. Dereplication analysis from MixONat, structure of top 50 metabolites: compounds S**41**–S**50** in DB-1

Step1: Dereplication of Bioactive Methanolic Extract

Dereplication analysis from MixONat, structure of top 50 metabolites: compounds S**51**-S**100** in DB-2

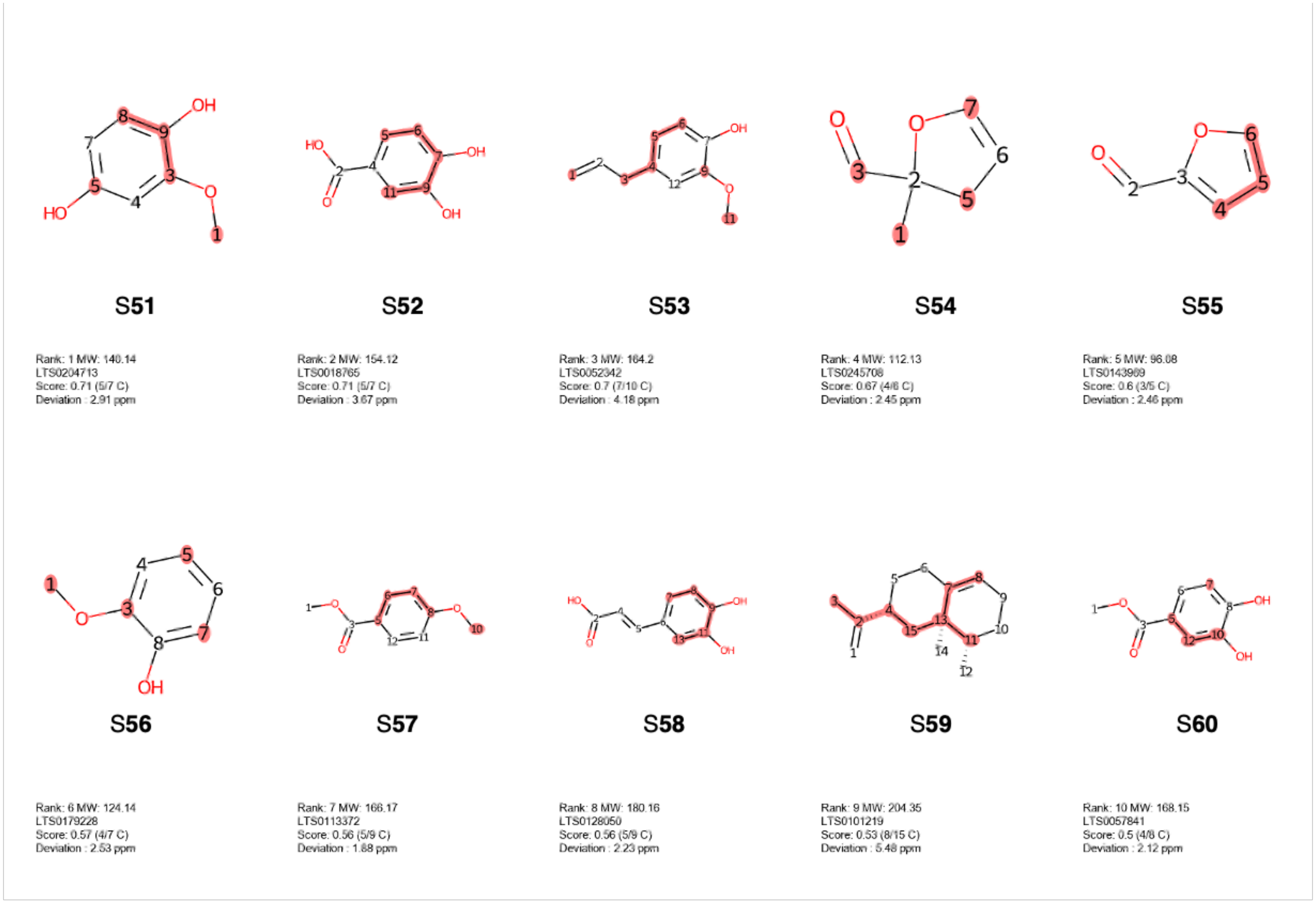

Figure S6. Dereplication analysis from MixONat, structure of top 50 metabolites: compounds S**51**–S**60** in DB-2

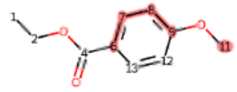

S61

Rank: 11 MW: 180.2
LTS0184041
Score: 0.5 (5/10 C)
Deviation : 2.28 ppm

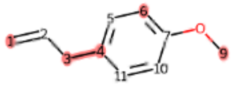

S62

Rank: 12 MW: 148.2
LTS0245226
Score: 0.5 (5/10 C)
Deviation : 2.49 ppm

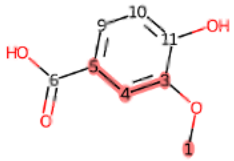

S63

Rank: 13 MW: 168.15
LTS0229113
Score: 0.5 (4/8 C)
Deviation : 2.93 ppm

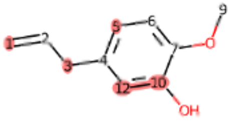

S64

Rank: 14 MW: 164.2
LTS0244260
Score: 0.5 (5/10 C)
Deviation : 3.6 ppm

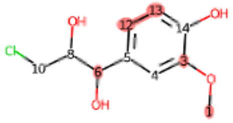

S65

Rank: 15 MW: 232.66
LTS0132524
Score: 0.5 (5/10 C)
Deviation : 3.82 ppm

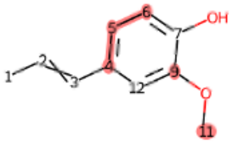

S66

Rank: 16 MW: 164.2
LTS0086128
Score: 0.5 (5/10 C)
Deviation : 4.19 ppm

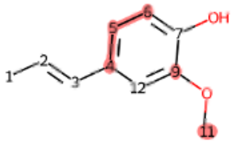

S67

Rank: 17 MW: 164.2
LTS0136836
Score: 0.5 (5/10 C)
Deviation : 4.19 ppm

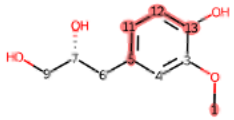

S68

Rank: 18 MW: 198.22
LTS0114565
Score: 0.5 (5/10 C)
Deviation : 4.22 ppm

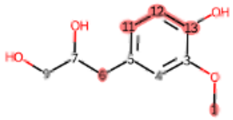

S69

Rank: 19 MW: 198.22
LTS0016014
Score: 0.5 (5/10 C)
Deviation : 4.23 ppm

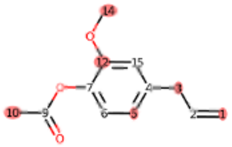

S70

Rank: 20 MW: 206.24
LTS0165886
Score: 0.5 (6/12 C)
Deviation : 4.26 ppm

Figure S7. Dereplication analysis from MixONat, structure of top 50 metabolites: compounds S**61**–S**70** in DB-2

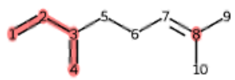

S71

Rank: 21 MW: 136.23
LTS0115731
Score: 0.5 (5/10 C)
Deviation : 4.28 ppm

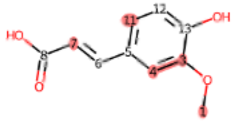

S72

Rank: 22 MW: 194.18
LTS0077326
Score: 0.5 (5/10 C)
Deviation : 4.43 ppm

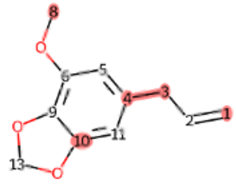

S73

Rank: 23 MW: 192.21
LTS0180101
Score: 0.45 (5/11 C)
Deviation : 2.21 ppm

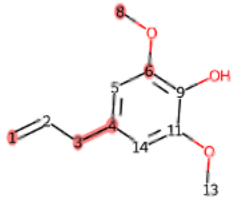

S74

Rank: 24 MW: 194.23
LTS0015297
Score: 0.45 (5/11 C)
Deviation : 3.16 ppm

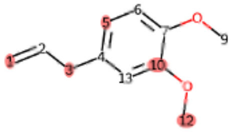

S75

Rank: 25 MW: 178.23
LTS0098881
Score: 0.45 (5/11 C)
Deviation : 3.96 ppm

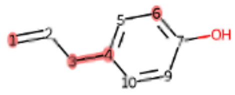

S76

Rank: 26 MW: 134.18
LTS0008864
Score: 0.44 (4/9 C)
Deviation : 3.53 ppm

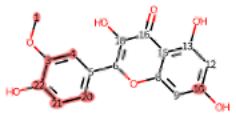

S77

Rank: 27 MW: 316.26
LTS0107505
Score: 0.44 (7/16 C)
Deviation : 3.98 ppm

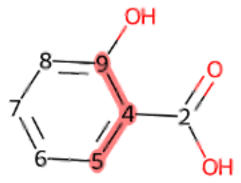

S78

Rank: 28 MW: 138.12
LTS0116548
Score: 0.43 (3/7 C)
Deviation : 1.14 ppm

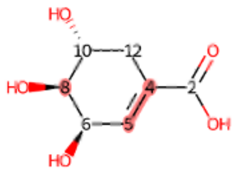

S79

Rank: 29 MW: 174.15
LTS0003899
Score: 0.43 (3/7 C)
Deviation : 1.85 ppm

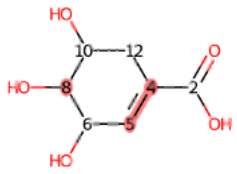

S80

Rank: 30 MW: 174.15
LTS0197942
Score: 0.43 (3/7 C)
Deviation : 1.85 ppm

Figure S8. Dereplication analysis from MixONat, structure of top 50 metabolites: compounds S**71**–S**80** in DB-2

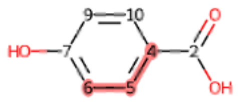

S81

Rank: 31 MW: 138.12
LTS0263634
Score: 0.43 (3/7 C)
Deviation : 2.05 ppm

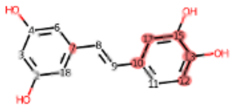

S82

Rank: 32 MW: 244.24
LTS0129442
Score: 0.43 (6/14 C)
Deviation : 2.74 ppm

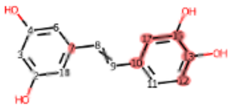

S83

Rank: 33 MW: 244.24
LTS0094372
Score: 0.43 (6/14 C)
Deviation : 2.74 ppm

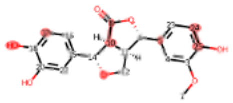

S84

Rank: 34 MW: 358.34
LTS0199807
Score: 0.42 (8/19 C)
Deviation : 3.98 ppm

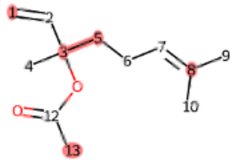

S85

Rank: 35 MW: 196.29
LTS0167325
Score: 0.42 (5/12 C)
Deviation : 1.75 ppm

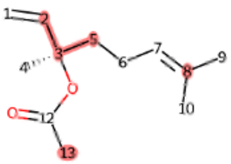

S86

Rank: 36 MW: 196.29
LTS0020520
Score: 0.42 (5/12 C)
Deviation : 2.42 ppm

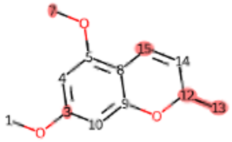

S87

Rank: 37 MW: 206.24
LTS0165858
Score: 0.42 (5/12 C)
Deviation : 3.36 ppm

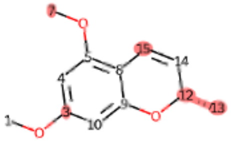

S88

Rank: 38 MW: 206.24
LTS0220668
Score: 0.42 (5/12 C)
Deviation : 4.16 ppm

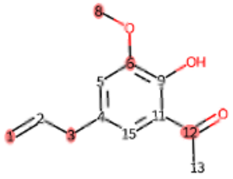

S89

Rank: 39 MW: 206.24
LTS0217393
Score: 0.42 (5/12 C)
Deviation : 4.37 ppm

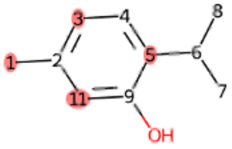

S90

Rank: 40 MW: 150.22
LTS0168527
Score: 0.4 (4/10 C)
Deviation : 0.93 ppm

Figure S9. Dereplication analysis from MixONat, structure of top 50 metabolites: compounds S**81**–S**90** in DB-2

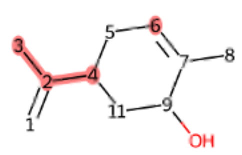

S91

Rank: 41 MW: 152.23
LTS0263183
Score: 0.4 (4/10 C)
Deviation : 1.18 ppm

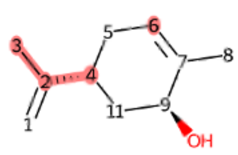

S92

Rank: 42 MW: 152.23
LTS0156471
Score: 0.4 (4/10 C)
Deviation : 1.48 ppm

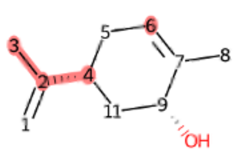

S93

Rank: 43 MW: 152.23
LTS0048903
Score: 0.4 (4/10 C)
Deviation : 1.48 ppm

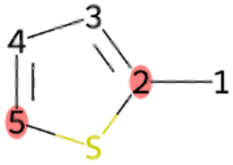

S94

Rank: 44 MW: 98.17
LTS0159316
Score: 0.4 (2/5 C)
Deviation : 1.52 ppm

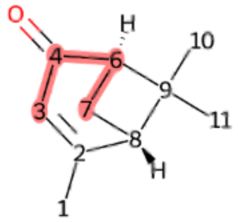

S95

Rank: 45 MW: 150.22
LTS0177954
Score: 0.4 (4/10 C)
Deviation : 1.65 ppm

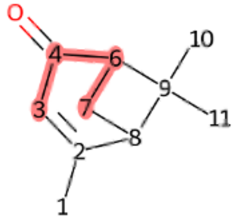

S96

Rank: 46 MW: 150.22
LTS0264577
Score: 0.4 (4/10 C)
Deviation : 1.65 ppm

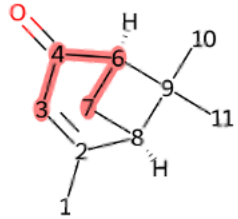

S97

Rank: 47 MW: 150.22
LTS0275336
Score: 0.4 (4/10 C)
Deviation : 1.65 ppm

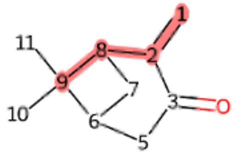

S98

Rank: 48 MW: 150.22
LTS0084836
Score: 0.4 (4/10 C)
Deviation : 1.85 ppm

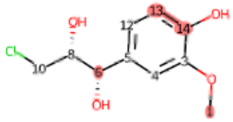

S99

Rank: 49 MW: 232.66
LTS0208953
Score: 0.4 (4/10 C)
Deviation : 1.89 ppm

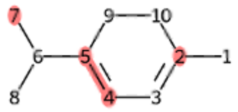

S100

Rank: 50 MW: 136.23
LTS0136858
Score: 0.4 (4/10 C)
Deviation : 1.95 ppm

Figure S10. Dereplication analysis from MixONat, structure of top 50 metabolites: compounds S**91**–S**100** in DB-2

Step1: Dereplication of Bioactive Methanolic extracts

Dereplication analysis from MixONat, structure of top 50 metabolites: compounds S**101**-S**150** in DB-3

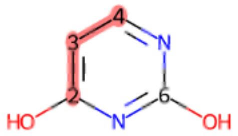

S101

Rank: 1 MW: 112.09
LTS0008205
Score: 0.75 (3/4 C)
Deviation : 0.95 ppm

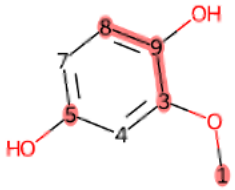

S102

Rank: 2 MW: 140.14
LTS0204713
Score: 0.71 (5/7 C)
Deviation : 2.91 ppm

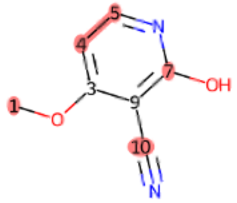

S103

Rank: 3 MW: 150.14
LTS0182439
Score: 0.71 (5/7 C)
Deviation : 3.17 ppm

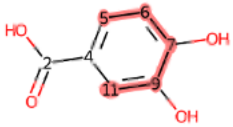

S104

Rank: 4 MW: 154.12
LTS0018765
Score: 0.71 (5/7 C)
Deviation : 3.67 ppm

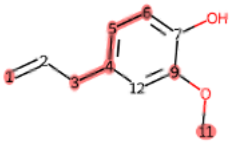

S105

Rank: 5 MW: 164.2
LTS0052342
Score: 0.7 (7/10 C)
Deviation : 4.18 ppm

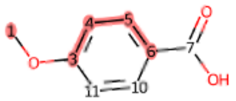

S106

Rank: 6 MW: 152.15
LTS0123492
Score: 0.62 (5/8 C)
Deviation : 1.92 ppm

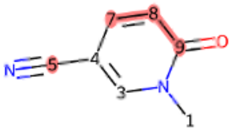

S107

Rank: 7 MW: 134.14
LTS0097756
Score: 0.57 (4/7 C)
Deviation : 1.88 ppm

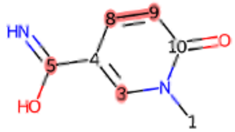

S108

Rank: 8 MW: 152.15
LTS0173819
Score: 0.57 (4/7 C)
Deviation : 2.04 ppm

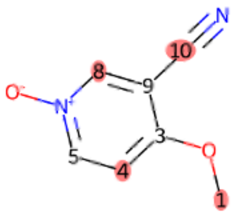

S109

Rank: 9 MW: 150.14
LTS0144355
Score: 0.57 (4/7 C)
Deviation : 3.07 ppm

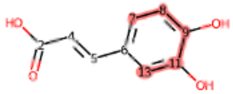

S110

Rank: 10 MW: 180.16
LTS0128050
Score: 0.56 (5/9 C)
Deviation : 2.23 ppm

Figure S11. Dereplication analysis from MixONat, structure of top 50 metabolites: compounds S**101**–S**110** in DB-3

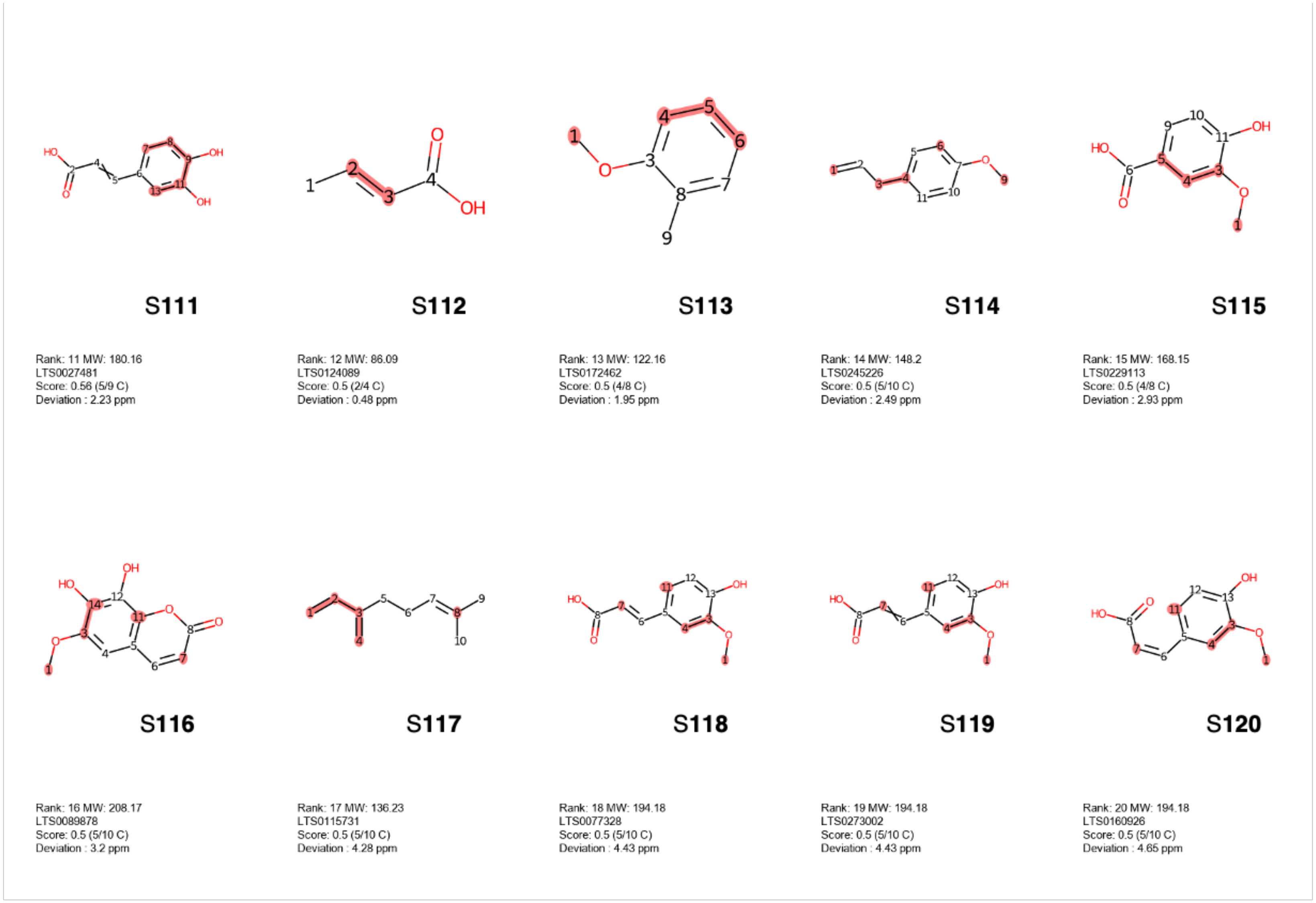

Figure S12. Dereplication analysis from MixONat, structure of top 50 metabolites: compounds S**111**–S**120** in DB-3

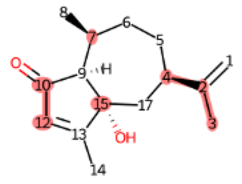

S121

Rank: 21 MW: 234.33
LTS0063360
Score: 0.47 (7/15 C)
Deviation : 5.46 ppm

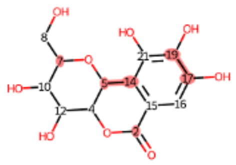

S122

Rank: 22 MW: 314.25
LTS0199880
Score: 0.46 (6/13 C)
Deviation : 3.05 ppm

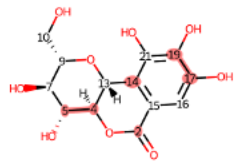

S123

Rank: 23 MW: 314.25
LTS0002782
Score: 0.46 (6/13 C)
Deviation : 3.1 ppm

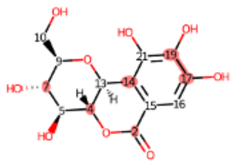

S124

Rank: 24 MW: 314.25
LTS0253078
Score: 0.46 (6/13 C)
Deviation : 3.38 ppm

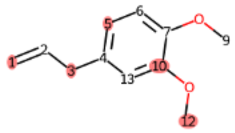

S125

Rank: 25 MW: 178.23
LTS0098881
Score: 0.45 (5/11 C)
Deviation : 3.96 ppm

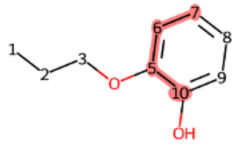

S126

Rank: 26 MW: 152.19
LTS0202481
Score: 0.44 (4/9 C)
Deviation : 1.97 ppm

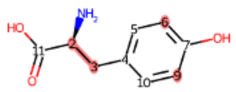

S127

Rank: 27 MW: 181.19
LTS0029981
Score: 0.44 (4/9 C)
Deviation : 2.43 ppm

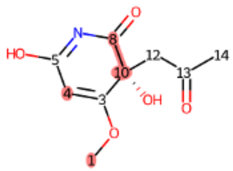

S128

Rank: 28 MW: 213.19
LTS0139506
Score: 0.44 (4/9 C)
Deviation : 2.6 ppm

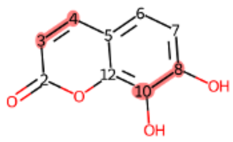

S129

Rank: 29 MW: 178.14
LTS0160083
Score: 0.44 (4/9 C)
Deviation : 3.18 ppm

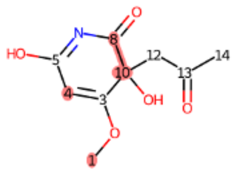

S130

Rank: 30 MW: 213.19
LTS0263607
Score: 0.44 (4/9 C)
Deviation : 3.34 ppm

Figure S13. Dereplication analysis from MixONat, structure of top 50 metabolites: compounds S**121**–S**130** in DB-3

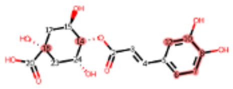

S131

Rank: 31 MW: 354.31
LTS0252404
Score: 0.44 (7/16 C)
Deviation : 2.58 ppm

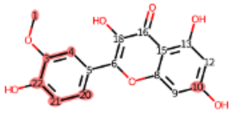

S132

Rank: 32 MW: 316.26
LTS0107505
Score: 0.44 (7/16 C)
Deviation : 3.98 ppm

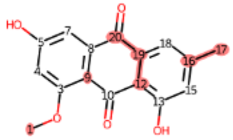

S133

Rank: 33 MW: 284.26
LTS0176534
Score: 0.44 (7/16 C)
Deviation : 4.75 ppm

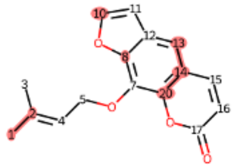

S134

Rank: 34 MW: 270.28
LTS0113114
Score: 0.44 (7/16 C)
Deviation : 4.86 ppm

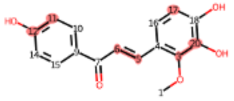

S135

Rank: 35 MW: 286.28
LTS0192338
Score: 0.44 (7/16 C)
Deviation : 4.97 ppm

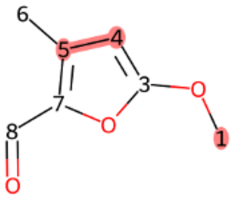

S136

Rank: 36 MW: 140.14
LTS0111119
Score: 0.43 (3/7 C)
Deviation : 1.02 ppm

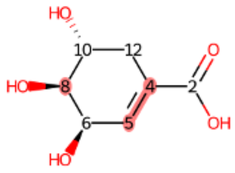

S137

Rank: 37 MW: 174.15
LTS0003899
Score: 0.43 (3/7 C)
Deviation : 1.85 ppm

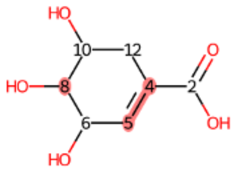

S138

Rank: 38 MW: 174.15
LTS0197942
Score: 0.43 (3/7 C)
Deviation : 1.85 ppm

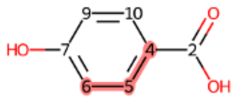

S139

Rank: 39 MW: 138.12
LTS0263634
Score: 0.43 (3/7 C)
Deviation : 2.05 ppm

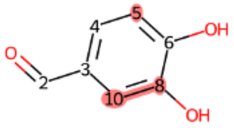

S140

Rank: 40 MW: 138.12
LTS0251601
Score: 0.43 (3/7 C)
Deviation : 2.49 ppm

Figure 14. Dereplication analysis from MixONat, structure of top 50 metabolites: compounds S**131**–S**140** in DB-3

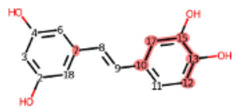

S141

Rank: 41 MW: 244.24
LTS0129442
Score: 0.43 (6/14 C)
Deviation : 2.74 ppm

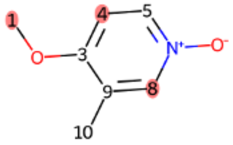

S142

Rank: 42 MW: 139.15
LTS0000093
Score: 0.43 (3/7 C)
Deviation : 3.03 ppm

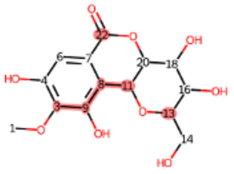

S143

Rank: 43 MW: 328.27
LTS0089177
Score: 0.43 (6/14 C)
Deviation : 3.66 ppm

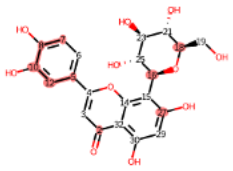

S144

Rank: 44 MW: 448.38
LTS0172349
Score: 0.43 (9/21 C)
Deviation : 5.37 ppm

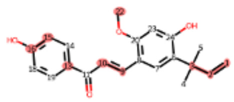

S145

Rank: 45 MW: 338.4
LTS0018907
Score: 0.43 (9/21 C)
Deviation : 6.4 ppm

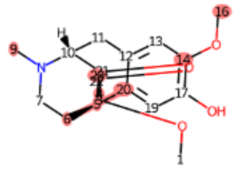

S146

Rank: 46 MW: 327.38
LTS0062940
Score: 0.42 (8/19 C)
Deviation : 3.96 ppm

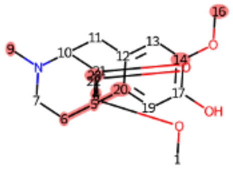

S147

Rank: 47 MW: 327.38
LTS0241076
Score: 0.42 (8/19 C)
Deviation : 3.96 ppm

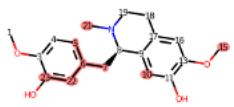

S148

Rank: 48 MW: 329.39
LTS0142027
Score: 0.42 (8/19 C)
Deviation : 4.4 ppm

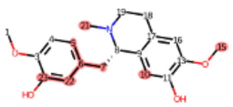

S149

Rank: 49 MW: 329.39
LTS0181650
Score: 0.42 (8/19 C)
Deviation : 4.4 ppm

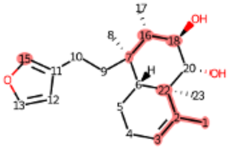

S150

Rank: 50 MW: 318.45
LTS0172264
Score: 0.42 (8/19 C)
Deviation : 5.77 ppm

Figure S15. Dereplication analysis from MixONat, structure of top 50 metabolites: compounds S**141**–S**150** in DB-3

Step1: Dereplication of bioactive fractions

A. F2-D1 (20) S**151**–S**170**

Figure S16: Dereplication analysis of F2-D1 from MixONat, structure of top 20 metabolites: compounds S**151**–S**160** from DB1;

Figure S17: Dereplication analysis of F2-D1 from MixONat, structure of top 20 metabolites: compounds S**161**–S**170** from DB1;

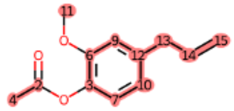

S151

Rank: 1 MW: 206.24
Eugenol acetate
Score: 1.0 (12/12 C)
Deviation : 2.56 ppm

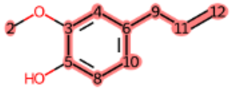

S152

Rank: 2 MW: 164.2
p-Eugenol
Score: 1.0 (10/10 C)
Deviation : 5.58 ppm

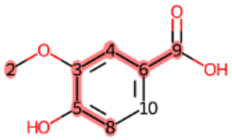

S153

Rank: 3 MW: 168.15
Vanillic acid
Score: 0.88 (7/8 C)
Deviation : 2.98 ppm

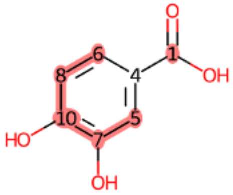

S154

Rank: 4 MW: 154.12
Protocatechuic acid
Score: 0.86 (6/7 C)
Deviation : 3.85 ppm

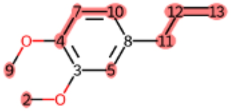

S155

Rank: 5 MW: 178.23
O-Methyleugenol
Score: 0.82 (9/11 C)
Deviation : 2.9 ppm

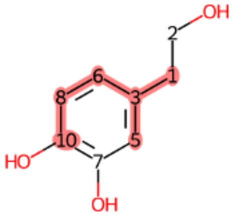

S156

Rank: 6 MW: 154.16
3-Hydroxytyrosol
Score: 0.75 (6/8 C)
Deviation : 2.47 ppm

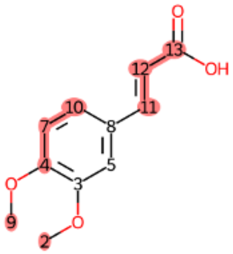

S157

Rank: 7 MW: 208.21
Methylferulic acid
Score: 0.73 (8/11 C)
Deviation : 3.8 ppm

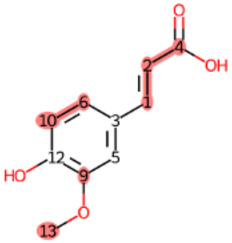

S158

Rank: 8 MW: 194.18
Ferulic acid
Score: 0.7 (7/10 C)
Deviation : 1.78 ppm

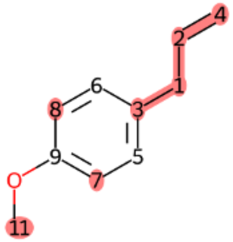

S159

Rank: 9 MW: 148.2
Estragole
Score: 0.7 (7/10 C)
Deviation : 4.23 ppm

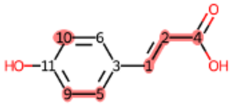

S160

Rank: 10 MW: 164.16
p-Coumaric acid
Score: 0.67 (6/9 C)
Deviation : 2.01 ppm

Figure S16. Dereplication analysis from MixONat, structure of top 50 metabolites: compounds S**151**–S**160** in DB-1

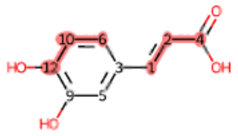

S161

Rank: 11 MW: 180.16
Caffeic acid
Score: 0.67 (6/9 C)
Deviation : 2.68 ppm

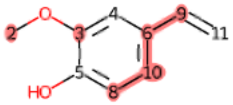

S162

Rank: 12 MW: 150.17
p-Vinylguaiacol
Score: 0.67 (6/9 C)
Deviation : 4.06 ppm

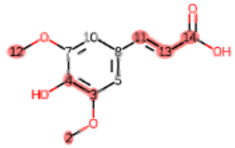

S163

Rank: 13 MW: 224.21
Sinapic acid
Score: 0.64 (7/11 C)
Deviation : 2.39 ppm

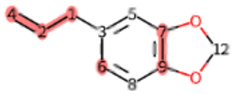

S164

Rank: 14 MW: 162.19
Shikimole
Score: 0.6 (6/10 C)
Deviation : 2.32 ppm

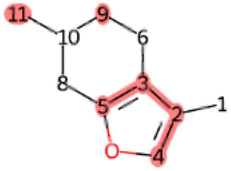

S165

Rank: 15 MW: 150.22
Menthofurane
Score: 0.6 (6/10 C)
Deviation : 2.34 ppm

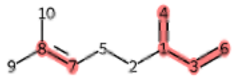

S166

Rank: 16 MW: 136.23
beta-Myrcene
Score: 0.6 (6/10 C)
Deviation : 3.18 ppm

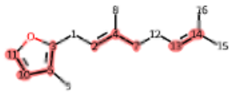

S167

Rank: 17 MW: 218.33
Sesquirose furan
Score: 0.6 (9/15 C)
Deviation : 4.82 ppm

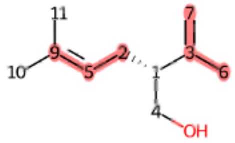

S168

Rank: 18 MW: 154.25
(-)-Lavandulol
Score: 0.6 (6/10 C)
Deviation : 4.92 ppm

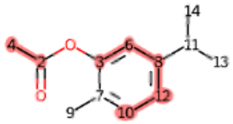

S169

Rank: 19 MW: 192.25
Carvacryl acetate
Score: 0.58 (7/12 C)
Deviation : 3.24 ppm

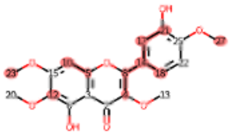

S170

Rank: 20 MW: 374.34
Casticin
Score: 0.58 (11/19 C)
Deviation : 5.86 ppm

Figure S17. Dereplication analysis from MixONat, structure of top 50 metabolites: compounds S**161**–S**170** in DB-1

Step1: Dereplication of bioactive fractions

B. F2-D2 (20) S**171**–S**190**

Figure S18: Dereplication analysis of F2-D2 from MixONat, structure of top 50 metabolites: compounds S**171**–S**180** from DB2.

Figure S19: Dereplication analysis from MixONat, structure of the top 50 metabolites: compounds S**181**–S**190** from DB2.

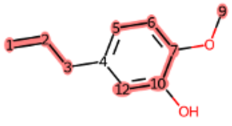

S171

Rank: 1 MW: 164.2
LTS0244260
Score: 0.9 (9/10 C)
Deviation : 3.79 ppm

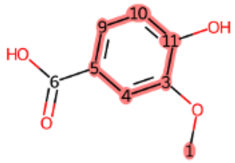

S172

Rank: 2 MW: 168.15
LTS0229113
Score: 0.88 (7/8 C)
Deviation : 3.94 ppm

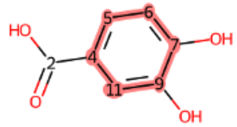

S173

Rank: 3 MW: 154.12
LTS0018765
Score: 0.86 (6/7 C)
Deviation : 3.94 ppm

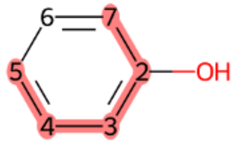

S174

Rank: 4 MW: 94.11
LTS0092642
Score: 0.83 (5/6 C)
Deviation : 2.66 ppm

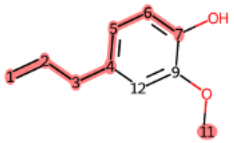

S175

Rank: 5 MW: 164.2
LTS0052342
Score: 0.8 (8/10 C)
Deviation : 4.08 ppm

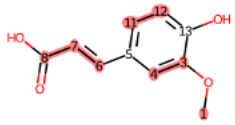

S176

Rank: 6 MW: 194.18
LTS0077328
Score: 0.8 (8/10 C)
Deviation : 4.8 ppm

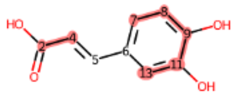

S177

Rank: 7 MW: 180.16
LTS0128050
Score: 0.78 (7/9 C)
Deviation : 4.32 ppm

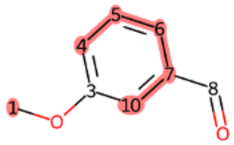

S178

Rank: 8 MW: 136.15
LTS0124278
Score: 0.75 (6/8 C)
Deviation : 3.44 ppm

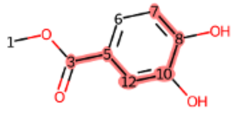

S179

Rank: 9 MW: 168.15
LTS0057841
Score: 0.75 (6/8 C)
Deviation : 3.88 ppm

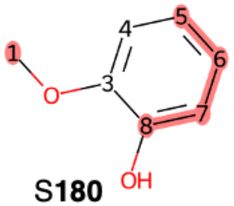

S180

Rank: 10 MW: 124.14
LTS0179228
Score: 0.71 (5/7 C)
Deviation : 2.7 ppm

Figure S18. Dereplication analysis from MixONat, structure of top 50 metabolites: compounds S**171**–S**180** in DB-2

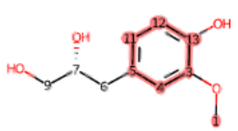

S181

Rank: 11 MW: 198.22
LTS0114565
Score: 0.7 (7/10 C)
Deviation : 3.01 ppm

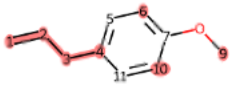

S182

Rank: 12 MW: 148.2
LTS0245226
Score: 0.7 (7/10 C)
Deviation : 3.56 ppm

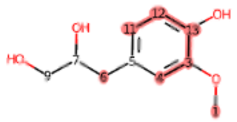

S183

Rank: 13 MW: 198.22
LTS0016014
Score: 0.7 (7/10 C)
Deviation : 3.84 ppm

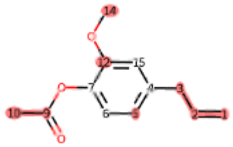

S184

Rank: 14 MW: 206.24
LTS0165886
Score: 0.67 (8/12 C)
Deviation : 1.61 ppm

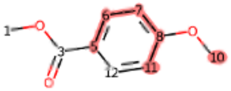

S185

Rank: 15 MW: 166.17
LTS0113372
Score: 0.67 (6/9 C)
Deviation : 3.24 ppm

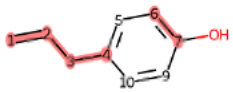

S186

Rank: 16 MW: 134.18
LTS0008864
Score: 0.67 (6/9 C)
Deviation : 3.43 ppm

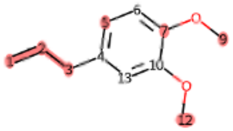

S187

Rank: 17 MW: 178.23
LTS0098881
Score: 0.64 (7/11 C)
Deviation : 1.89 ppm

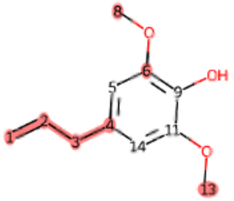

S188

Rank: 18 MW: 194.23
LTS0015297
Score: 0.64 (7/11 C)
Deviation : 2.45 ppm

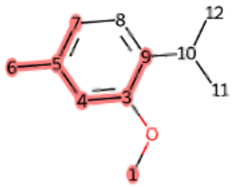

S189

Rank: 19 MW: 164.24
LTS0054789
Score: 0.64 (7/11 C)
Deviation : 4.74 ppm

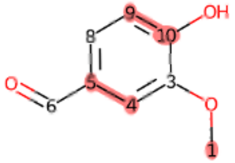

S190

Rank: 20 MW: 152.15
LTS0136163
Score: 0.62 (5/8 C)
Deviation : 2.95 ppm

Figure S19. Dereplication analysis from MixONat, structure of top 50 metabolites: compounds S**181**–S**190** in DB-2

Step1: Dereplication of bioactive fractions

C. F12-D1 (20) S**191**–S**210**

Figure S20: Dereplication analysis of F12-D1 from MixONat, structure of the top 50 metabolites: compounds S**191**–S**200** from D1.

Figure S21: Dereplication analysis from MixONat, structure of the top 50 metabolites: compounds S**201**–S**210** from D1.

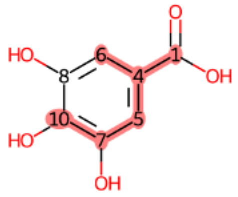

S191

Rank: 1 MW: 170.12
Gallic acid
Score: 0.86 (6/7 C)
Deviation : 5.04 ppm

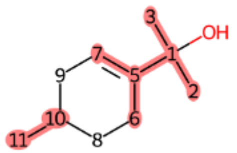

S192

Rank: 2 MW: 154.25
p-Menth-3-en-8-ol
Score: 0.8 (8/10 C)
Deviation : 3.88 ppm

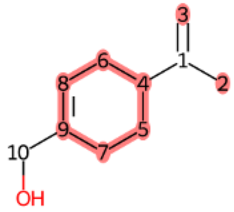

S193

Rank: 3 MW: 152.23
Perillol
Score: 0.8 (8/10 C)
Deviation : 5.35 ppm

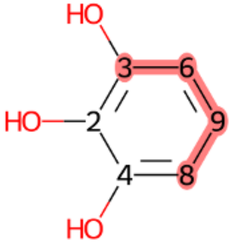

S194

Rank: 4 MW: 126.11
1,2,3-Benzenetriol
Score: 0.67 (4/6 C)
Deviation : 0.71 ppm

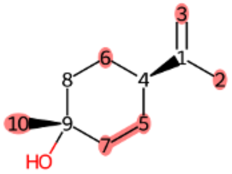

S195

Rank: 5 MW: 154.25
cis-beta-Terpineol
Score: 0.6 (6/10 C)
Deviation : 1.16 ppm

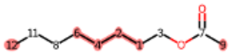

S196

Rank: 6 MW: 172.26
1-Acetoxyoctane
Score: 0.6 (6/10 C)
Deviation : 1.7 ppm

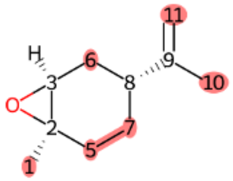

S197

Rank: 7 MW: 152.23
cis-Limonene epoxide
Score: 0.6 (6/10 C)
Deviation : 2.76 ppm

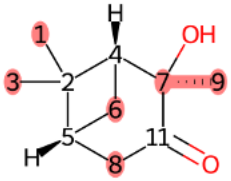

S198

Rank: 8 MW: 168.23
CAS-24047-72-1
Score: 0.6 (6/10 C)
Deviation : 2.8 ppm

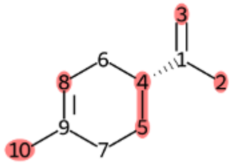

S199

Rank: 9 MW: 136.23
(+)-Limonene
Score: 0.6 (6/10 C)
Deviation : 2.89 ppm

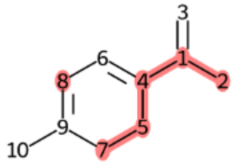

S200

Rank: 10 MW: 134.22
p-Menta-1,3,8-triene
Score: 0.6 (6/10 C)
Deviation : 2.97 ppm

Figure S20. Dereplication analysis from MixONat, structure of top 50 metabolites: compounds S**191**–S**200** in DB-1

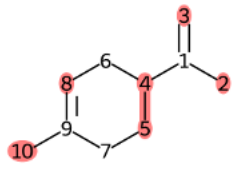

S201

Rank: 11 MW: 136.23
Limonene
Score: 0.6 (6/10 C)
Deviation : 3.09 ppm

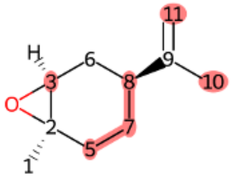

S202

Rank: 12 MW: 152.23
trans-Limonene epoxide
Score: 0.6 (6/10 C)
Deviation : 3.1 ppm

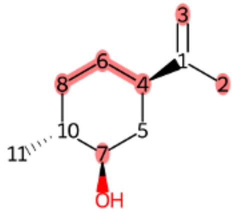

S203

Rank: 13 MW: 154.25
Dihydrocarveol
Score: 0.6 (6/10 C)
Deviation : 3.59 ppm

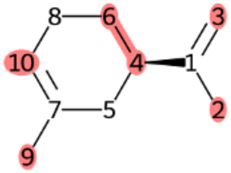

S204

Rank: 14 MW: 136.23
Sylvestrene
Score: 0.6 (6/10 C)
Deviation : 3.84 ppm

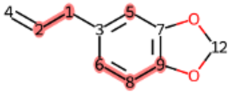

S205

Rank: 15 MW: 162.19
Shikimole
Score: 0.6 (6/10 C)
Deviation : 4.4 ppm

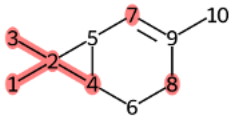

S206

Rank: 16 MW: 136.23
2-Carene
Score: 0.6 (6/10 C)
Deviation : 4.52 ppm

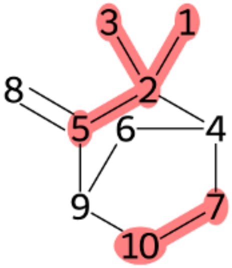

S207

Rank: 17 MW: 136.23
Camphene
Score: 0.6 (6/10 C)
Deviation : 5.45 ppm

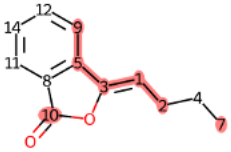

S208

Rank: 18 MW: 188.22
3-Butylidenephthalide
Score: 0.58 (7/12 C)
Deviation : 4.85 ppm

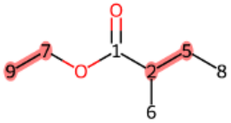

S209

Rank: 19 MW: 130.18
Ethyl 2-methylbutyrate
Score: 0.57 (4/7 C)
Deviation : 1.35 ppm

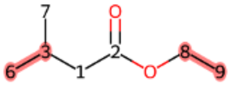

S210

Rank: 20 MW: 130.18
Ethyl isopentanoate
Score: 0.57 (4/7 C)
Deviation : 2.84 ppm

Figure S21. Dereplication analysis from MixONat, structure of top 50 metabolites: compounds S**201**–S**210** in DB-1

Step1: Dereplication of bioactive fractions

D. F12-D2 (20) S**211**–S**230**

Figure S22: Dereplication analysis of F12-D2 from MixONat, structure of the top 50 metabolites: compounds S**211**–S**220** from DB2.

Figure S23: Dereplication analysis from MixONat, structure of the top 50 metabolites: compounds S**221**–S**230** from DB2.

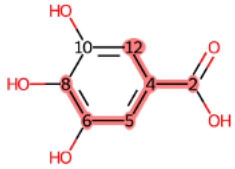

S211

Rank: 1 MW: 170.12
LTS0222857
Score: 0.86 (6/7 C)
Deviation : 1.36 ppm

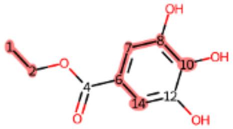

S212

Rank: 2 MW: 198.17
LTS0270645
Score: 0.78 (7/9 C)
Deviation : 3.15 ppm

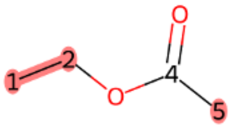

S213

Rank: 3 MW: 88.11
LTS0196824
Score: 0.75 (3/4 C)
Deviation : 0.46 ppm

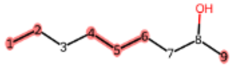

S214

Rank: 4 MW: 144.25
LTS0264829
Score: 0.67 (6/9 C)
Deviation : 3.27 ppm

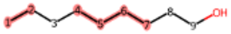

S215

Rank: 5 MW: 144.25
LTS0157379
Score: 0.67 (6/9 C)
Deviation : 3.52 ppm

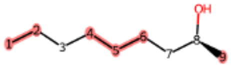

S216

Rank: 6 MW: 144.25
LTS0186068
Score: 0.67 (6/9 C)
Deviation : 3.54 ppm

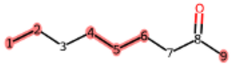

S217

Rank: 7 MW: 142.24
LTS0245014
Score: 0.67 (6/9 C)
Deviation : 3.81 ppm

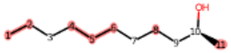

S218

Rank: 8 MW: 172.31
LTS0195028
Score: 0.64 (7/11 C)
Deviation : 4.07 ppm

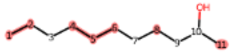

S219

Rank: 9 MW: 172.31
LTS0141928
Score: 0.64 (7/11 C)
Deviation : 4.07 ppm

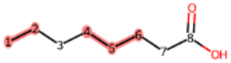

S220

Rank: 10 MW: 144.21
LTS0254176
Score: 0.62 (5/8 C)
Deviation : 2.0 ppm

Figure S22. Dereplication analysis from MixONat, structure of top 50 metabolites: compounds S**211**–S**220** in DB-2

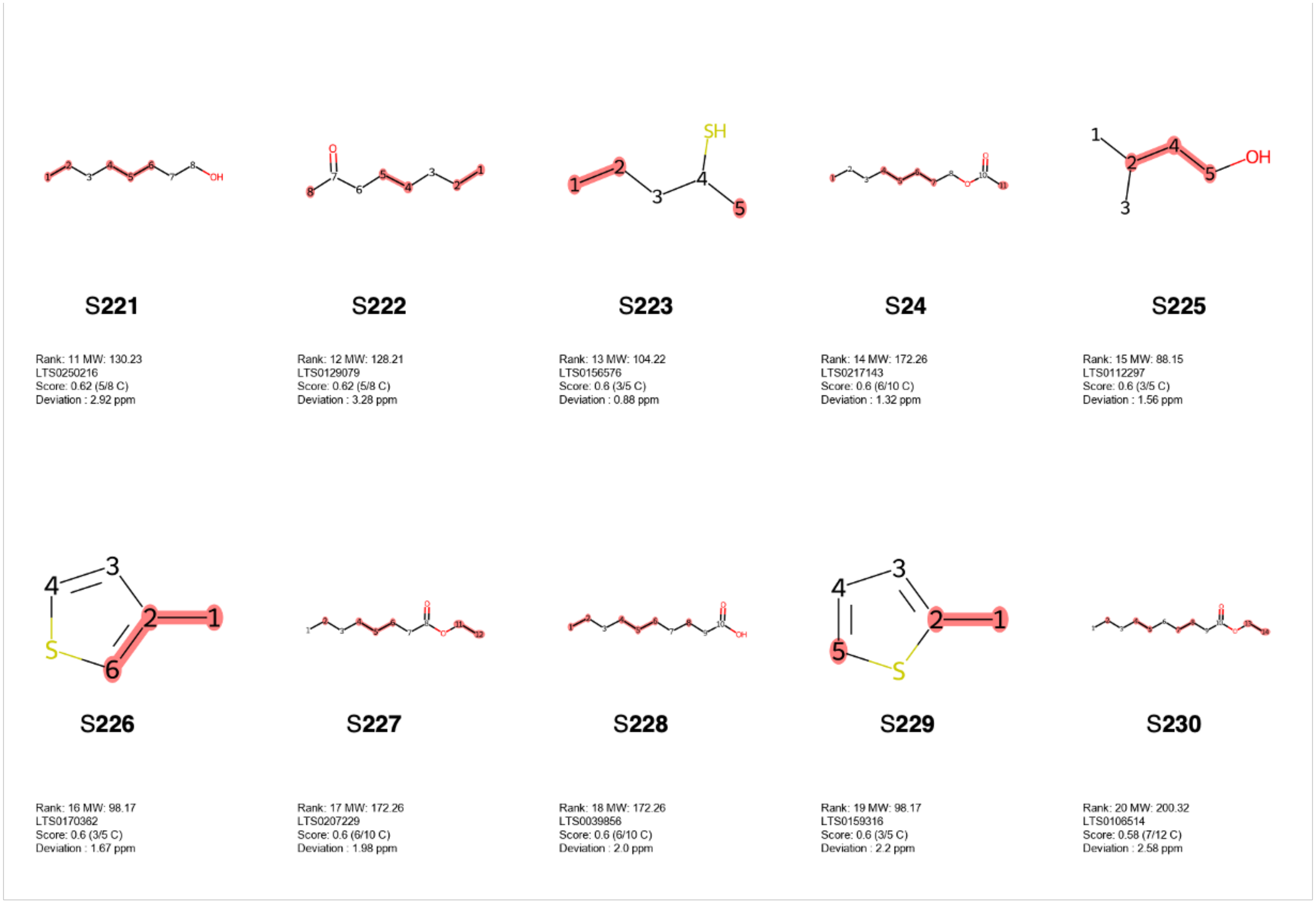

Figure S23. Dereplication analysis from MixONat, structure of top 50 metabolites: compounds S**221**–S**230** in DB-2

Step2: Dereplication **of** bioactive fractions

A. F2-D4 (20) S**231**–S**250**

Figure S24: Dereplication analysis of F2-DB4 from MixONat, structure of the top 50 metabolites: compounds S**231**–S**240** from DB-4.

Figure S25: Dereplication analysis from MixONat; structure of top 50 metabolites: compounds S**241**–S**250** from DB-4.

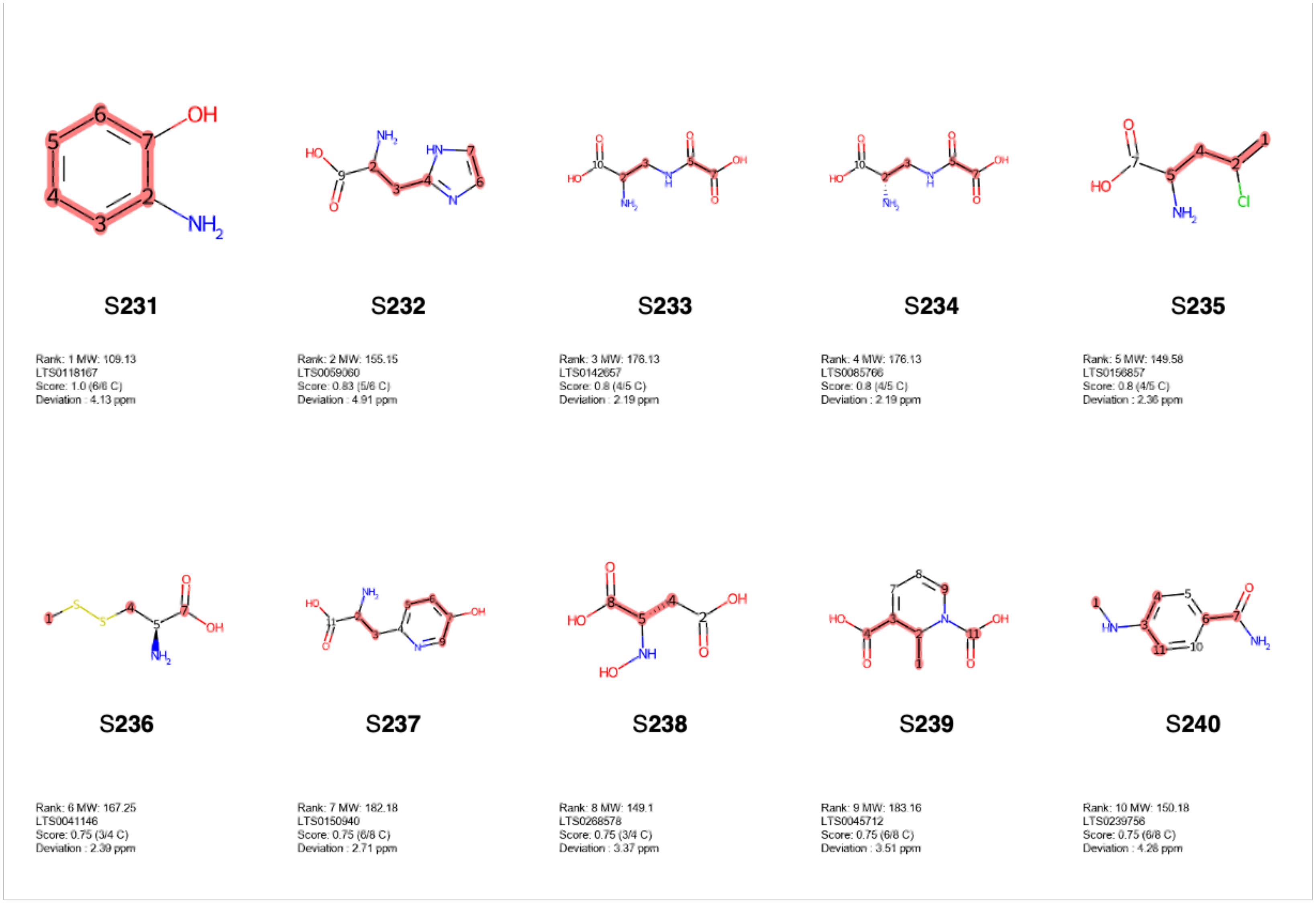

Figure S24. Dereplication analysis from MixONat, structure of top 50 metabolites: compounds S**231**–S**240** in DB-4

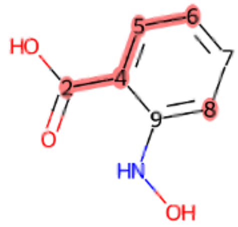

S241

Rank: 11 MW: 153.14
LTS0023452
Score: 0.71 (5/7 C)
Deviation : 2.21 ppm

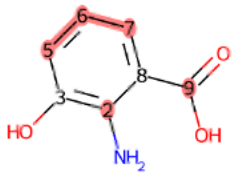

S242

Rank: 12 MW: 153.14
LTS0227876
Score: 0.71 (5/7 C)
Deviation : 3.0 ppm

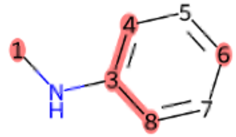

S243

Rank: 13 MW: 107.15
LTS0086729
Score: 0.71 (5/7 C)
Deviation : 3.38 ppm

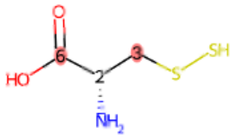

S244

Rank: 14 MW: 153.23
LTS0108890
Score: 0.67 (2/3 C)
Deviation : 1.3 ppm

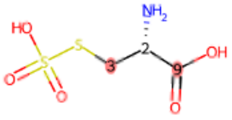

S245

Rank: 15 MW: 201.22
LTS0007830
Score: 0.67 (2/3 C)
Deviation : 1.3 ppm

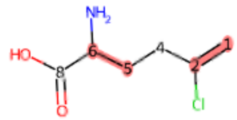

S246

Rank: 16 MW: 163.6
LTS0158860
Score: 0.67 (4/6 C)
Deviation : 1.76 ppm

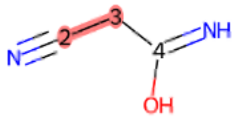

S247

Rank: 17 MW: 84.08
LTS0078751
Score: 0.67 (2/3 C)
Deviation : 2.21 ppm

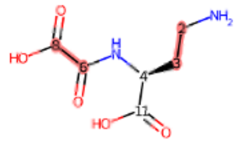

S248

Rank: 18 MW: 190.15
LTS0222131
Score: 0.67 (4/6 C)
Deviation : 2.4 ppm

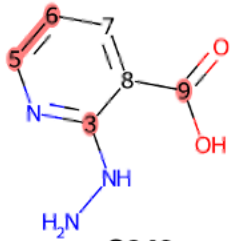

S249

Rank: 19 MW: 153.14
LTS0178548
Score: 0.67 (4/6 C)
Deviation : 2.55 ppm

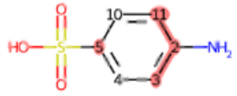

S250

Rank: 20 MW: 173.19
LTS0267393
Score: 0.67 (4/6 C)
Deviation : 2.67 ppm

Figure S25. Dereplication analysis from MixONat, structure of top 50 metabolites: compounds S**241**–S**250** in DB-4

Dereplication of bioactive fractions

Step2

B. F2-DB-5 (20) S**251**–S**270**

Figure S26: Dereplication analysis of F2-DB-5 from MixONat, structure of the top 50 metabolites: compounds S**251**–S**260** from DB5.

Figure S27: Dereplication analysis of F2-DB-5 from MixONat; structure of top 50 metabolites: compounds S**261**–S**270** from DB5.

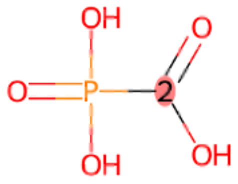

S251

Rank: 1 MW: 126.01
LTS0038082
Score: 1.0 (1/1 C)
Deviation : 0.56 ppm

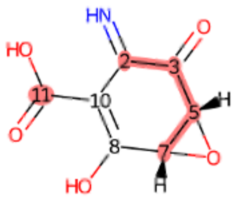

S252

Rank: 2 MW: 183.12
LTS0040576
Score: 0.71 (5/7 C)
Deviation : 4.69 ppm

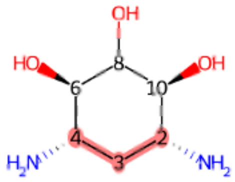

S253

Rank: 3 MW: 162.19
LTS0195613
Score: 0.5 (3/6 C)
Deviation : 1.76 ppm

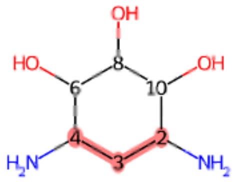

S254

Rank: 4 MW: 162.19
LTS0052927
Score: 0.5 (3/6 C)
Deviation : 1.76 ppm

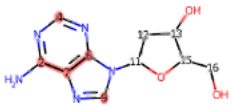

S255

Rank: 5 MW: 251.24
LTS0165339
Score: 0.5 (5/10 C)
Deviation : 3.87 ppm

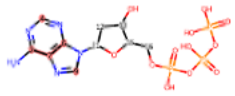

S256

Rank: 6 MW: 491.18
LTS0094381
Score: 0.5 (5/10 C)
Deviation : 3.87 ppm

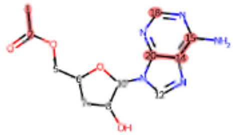

S257

Rank: 7 MW: 293.28
LTS0219693
Score: 0.5 (6/12 C)
Deviation : 5.19 ppm

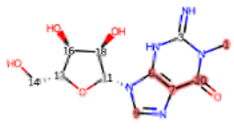

S258

Rank: 8 MW: 297.27
LTS0104993
Score: 0.45 (5/11 C)
Deviation : 2.58 ppm

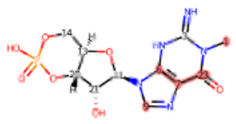

S259

Rank: 9 MW: 359.23
LTS0123889
Score: 0.45 (5/11 C)
Deviation : 2.58 ppm

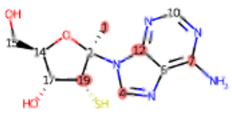

S260

Rank: 10 MW: 297.34
LTS0179693
Score: 0.45 (5/11 C)
Deviation : 4.1 ppm

Figure S26. Dereplication analysis from MixONat, structure of top 50 metabolites: compounds S**251**–S**260** in DB-5

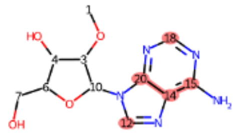

S261

Rank: 11 MW: 281.27
LTS0118558
Score: 0.45 (5/11 C)
Deviation : 4.14 ppm

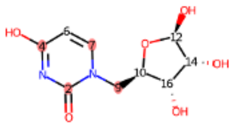

S262

Rank: 12 MW: 244.2
LTS0184396
Score: 0.44 (4/9 C)
Deviation : 2.35 ppm

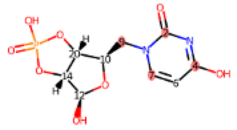

S263

Rank: 13 MW: 306.17
LTS0083926
Score: 0.44 (4/9 C)
Deviation : 2.35 ppm

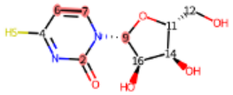

S264

Rank: 14 MW: 260.27
LTS0161655
Score: 0.44 (4/9 C)
Deviation : 2.55 ppm

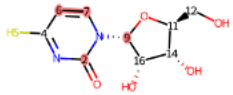

S265

Rank: 15 MW: 260.27
LTS0102288
Score: 0.44 (4/9 C)
Deviation : 2.99 ppm

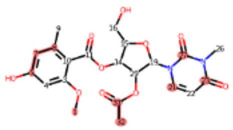

S266

Rank: 16 MW: 464.42
LTS0169315
Score: 0.43 (9/21 C)
Deviation : 5.4 ppm

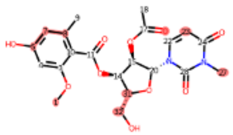

S267

Rank: 17 MW: 464.42
LTS0080446
Score: 0.43 (9/21 C)
Deviation : 6.18 ppm

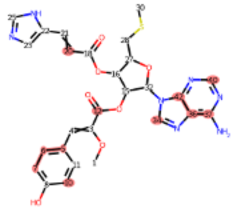

S268

Rank: 18 MW: 593.61
LTS0154418
Score: 0.42 (11/26 C)
Deviation : 8.09 ppm

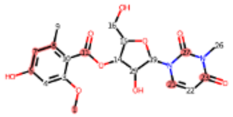

S269

Rank: 19 MW: 422.39
LTS0209848
Score: 0.42 (8/19 C)
Deviation : 5.59 ppm

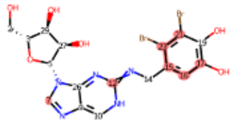

S270

Rank: 20 MW: 547.15
LTS0068413
Score: 0.41 (7/17 C)
Deviation : 3.33 ppm

Figure S27. Dereplication analysis from MixONat, structure of top 50 metabolites: compounds S**261**–S**270** in DB-5

Step2: Dereplication of bioactive fractions

C. F12-DB-4 (20) S**271**–S**290**

Figure S28: Dereplication analysis of F2-DB-4 from MixONat, structure of the top 50 metabolites: compounds S**271**–S**280** from DB-4.

Figure S29: Dereplication analysis of F2-DB-4 from MixONat; structure of top 50 metabolites: compounds S**281**–S**290** from DB-4.

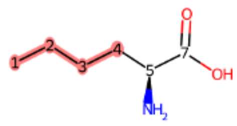

S271

Rank: 1 MW: 131.17
LTS0132632
Score: 0.67 (4/6 C)
Deviation : 3.03 ppm

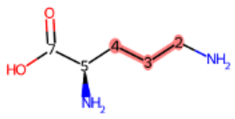

S272

Rank: 2 MW: 132.16
LTS0224949
Score: 0.6 (3/5 C)
Deviation : 1.57 ppm

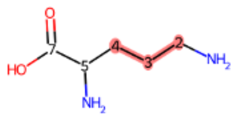

S273

Rank: 3 MW: 132.16
LTS0150033
Score: 0.6 (3/5 C)
Deviation : 1.57 ppm

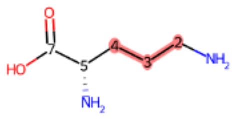

S274

Rank: 4 MW: 132.16
LTS0093444
Score: 0.6 (3/5 C)
Deviation : 1.57 ppm

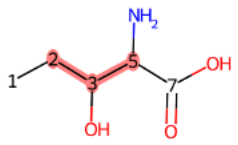

S275

Rank: 5 MW: 133.15
LTS0113357
Score: 0.6 (3/5 C)
Deviation : 2.47 ppm

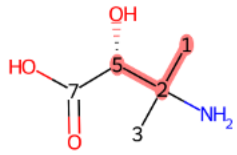

S276

Rank: 6 MW: 133.15
LTS0006181
Score: 0.6 (3/5 C)
Deviation : 2.55 ppm

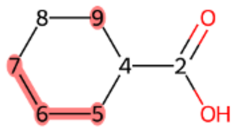

S277

Rank: 7 MW: 128.17
LTS0004486
Score: 0.57 (4/7 C)
Deviation : 1.12 ppm

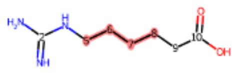

S278

Rank: 8 MW: 173.21
LTS0047822
Score: 0.57 (4/7 C)
Deviation : 1.19 ppm

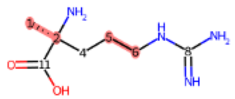

S279

Rank: 9 MW: 188.23
LTS0034990
Score: 0.57 (4/7 C)
Deviation : 1.36 ppm

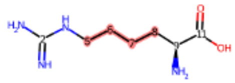

S280

Rank: 10 MW: 188.23
LTS0176041
Score: 0.57 (4/7 C)
Deviation : 1.66 ppm

Figure S28. Dereplication analysis from MixONat, structure of top 50 metabolites: compounds S**271**–S**280** in DB-4

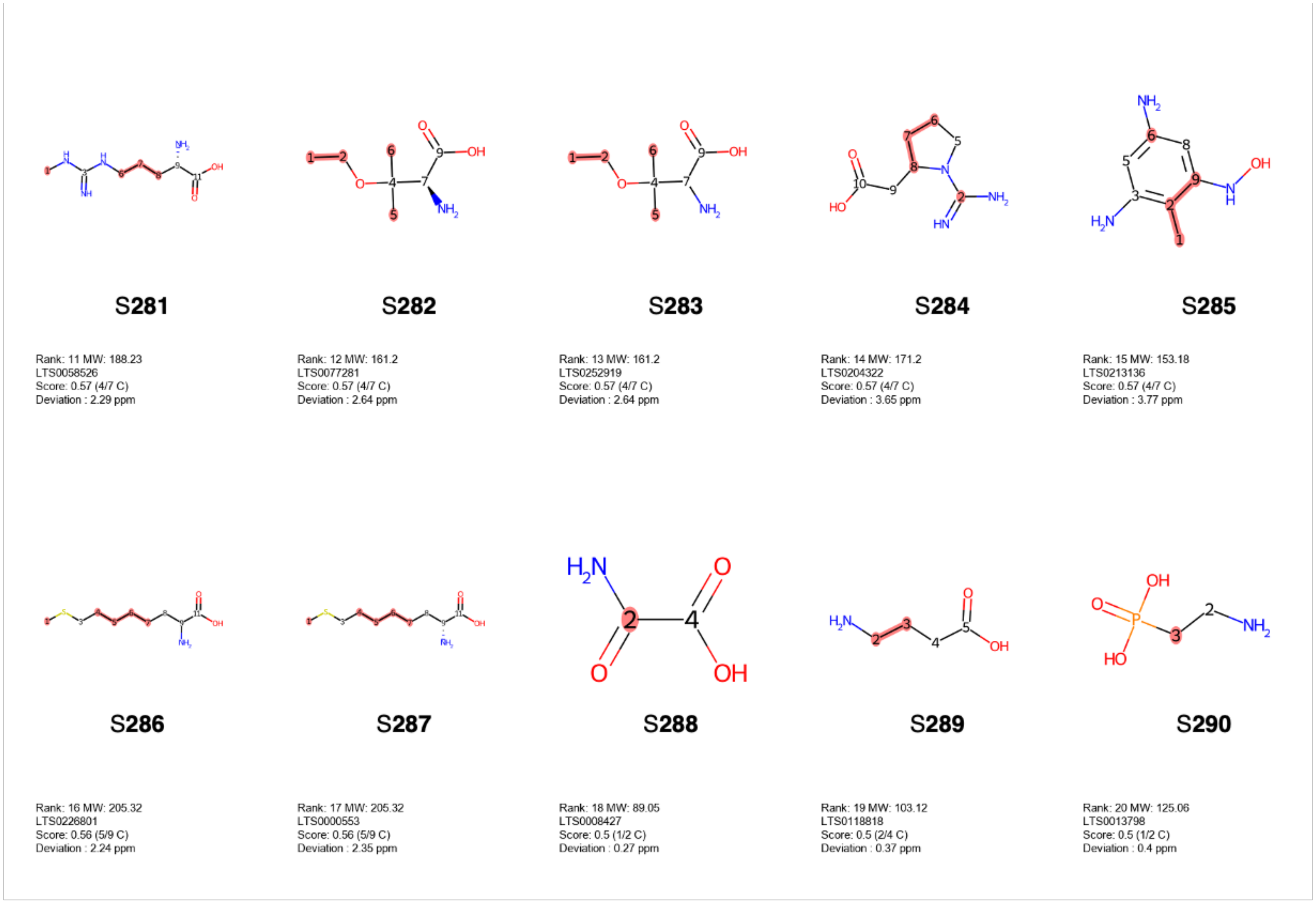

Figure S29. Dereplication analysis from MixONat, structure of top 50 metabolites: compounds S**281**–S**290** in DB-4

Step2: Dereplication of bioactive fractions

D. F12-DB5 (20) S**291**–S**310**

Figure S30: Dereplication analysis from MixONat, structure of the top 50 metabolites: compounds S**291**–S**300** from DB-5.

Figure S31: Dereplication analysis from MixONat; structure of top 50 metabolites: compounds S**300**–S**310** from DB-5.

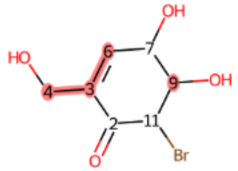

S291

Rank: 1 MW: 237.05
LTS0033735
Score: 0.57 (4/7 C)
Deviation : 2.6 ppm

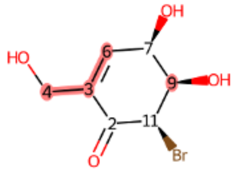

S292

Rank: 2 MW: 237.05
LTS0209560
Score: 0.57 (4/7 C)
Deviation : 2.7 ppm

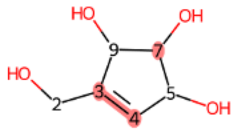

S293

Rank: 3 MW: 146.14
LTS0037932
Score: 0.5 (3/6 C)
Deviation : 0.8 ppm

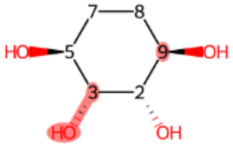

S294

Rank: 4 MW: 148.16
LTS0064982
Score: 0.5 (3/6 C)
Deviation : 1.09 ppm

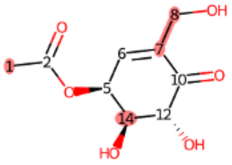

S295

Rank: 5 MW: 216.19
LTS0017548
Score: 0.44 (4/9 C)
Deviation : 3.06 ppm

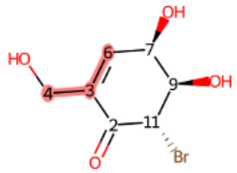

S296

Rank: 6 MW: 237.05
LTS0001351
Score: 0.43 (3/7 C)
Deviation : 2.06 ppm

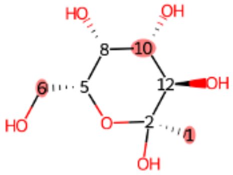

S297

Rank: 7 MW: 194.18
LTS0107432
Score: 0.43 (3/7 C)
Deviation : 2.44 ppm

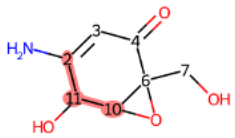

S298

Rank: 8 MW: 171.15
LTS0092769
Score: 0.43 (3/7 C)
Deviation : 2.66 ppm

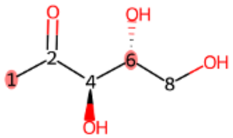

S299

Rank: 9 MW: 134.13
LTS0095924
Score: 0.4 (2/5 C)
Deviation : 0.51 ppm

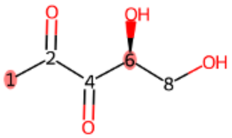

S300

Rank: 10 MW: 132.11
LTS0055321
Score: 0.4 (2/5 C)
Deviation : 0.59 ppm

Figure S30. Dereplication analysis from MixONat, structure of top 50 metabolites: compounds S**291**–S**300** in DB-5

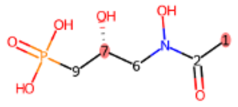

S301

Rank: 11 MW: 213.13
LTS0131789
Score: 0.4 (2/5 C)
Deviation : 0.61 ppm

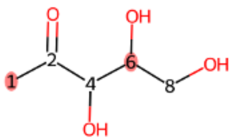

S302

Rank: 12 MW: 134.13
LTS0031280
Score: 0.4 (2/5 C)
Deviation : 0.7 ppm

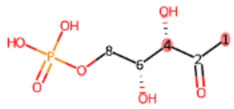

S303

Rank: 13 MW: 214.11
LTS0265924
Score: 0.4 (2/5 C)
Deviation : 1.09 ppm

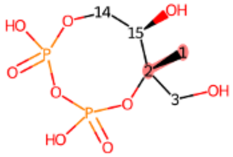

S304

Rank: 14 MW: 278.09
LTS0208549
Score: 0.4 (2/5 C)
Deviation : 1.19 ppm

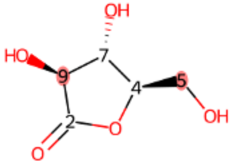

S305

Rank: 15 MW: 148.11
LTS0165092
Score: 0.4 (2/5 C)
Deviation : 1.21 ppm

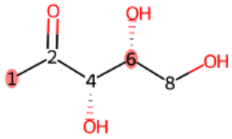

S306

Rank: 16 MW: 134.13
LTS0138977
Score: 0.4 (2/5 C)
Deviation : 1.31 ppm

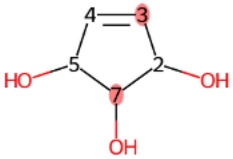

S307

Rank: 17 MW: 116.12
LTS0132061
Score: 0.4 (2/5 C)
Deviation : 1.58 ppm

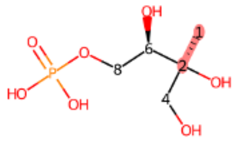

S308

Rank: 18 MW: 216.13
LTS0095389
Score: 0.4 (2/5 C)
Deviation : 1.59 ppm

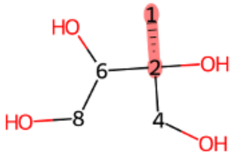

S309

Rank: 19 MW: 136.15
LTS0087898
Score: 0.4 (2/5 C)
Deviation : 1.59 ppm

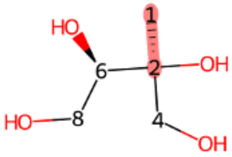

S310

Rank: 20 MW: 136.15
LTS0197229
Score: 0.4 (2/5 C)
Deviation : 1.59 ppm

Figure S31. Dereplication analysis from MixONat, structure of top 50 metabolites: compounds S**301**–S**310** in DB-5
